# Retinal Pigment Epithelium‐Targeting Gene Therapy Corrects Ocular Symptoms in Mouse and Rat Models of Oculocutaneous Albinism Type I

**DOI:** 10.1002/mco2.70433

**Published:** 2025-10-20

**Authors:** Li Song, Chengda Ren, Min Luo, Jing Su, Xiu Jin, Jiamei Fu, Qiuxia Xu, Xiaoyi Wu, Fanfei Liu, Qin Ye, Ming Hu, Man Liu, Qiqi Li, Yifang An, Manjun Li, Qingnan Wang, Kaiqin She, Fang Lu, Yang Yang

**Affiliations:** ^1^ State Key Laboratory of Biotherapy and Cancer Center West China Hospital Sichuan University and Collaborative Innovation Center Chengdu China; ^2^ Department of Ophthalmology West China Hospital Sichuan University Chengdu China; ^3^ Chengdu Genevector Therapeutics, Inc. Chengdu China

**Keywords:** gene therapy, OCA1, subretinal, suprachoroidal, RPE‐specific promoter

## Abstract

Oculocutaneous albinism (OCA) represents a genetically heterogeneous autosomal recessive condition marked by reduced melanin production in cutaneous and ocular tissues. This disorder primarily arises from mutations in the *TYR* gene, which encodes tyrosinase—the rate‐limiting enzyme in melanogenesis. Such genetic defects lead to impaired or absent tyrosinase activity, consequently causing melanin deficiency. Currently, no curative treatment exists for OCA1. In this study, we investigated the efficacy of AAV vector‐based gene therapy in two murine models of OCA1, evaluating its potential as a therapeutic intervention. B6 albino mice were injected with three AAV8 vectors containing distinct promoters at different dosages, among which AAV8.hRPE65p.hTYRco showed the best therapeutic effect at a dose of 1 × 10^9^ GC/eye. This RPE‐targeted strategy restored the expression of functional tyrosinase and melanin deposition in the RPE layer. More importantly, the retinal structure and visual function were maintained at nearly normal levels for up to 12 months, with no obvious toxicity. In addition, we demonstrated that the suprachoroidal cavity delivery of AAV8.hRPE65p.hTYRco restored the expression of tyrosinase and relieved ocular dysfunction in Wistar rats for at least 12 months. The results revealed a long‐term, effective, safe strategy for treating OCA1.

## Graphical abstract

RPE‐targeted gene therapy restored melanin expression and relieved ocular dysfunction. By optimizing the injection method, a wider range of melanin expression and better therapeutic effects have been achieved.

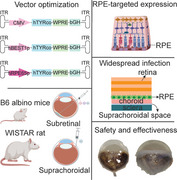

## 1 Introduction

Oculocutaneous albinism (OCA) is a heterogeneous autosomal recessive genetic disorder caused by dysfunctional melanin synthesis [1, 2]. OCA is classified into eight nonsyndromic forms (OCA1‐8) based on the gene in which it is mutated [3]. OCA1 is the most common type of OCA and accounts for 50% of all patients with OCA worldwide, with a prevalence of approximately 1/40,000 of the world's population [4]. OCA1 results from biallelic pathogenic variants in the *tyrosinase* (*TYR*) gene located at chromosome 11q14.3, which lead to impaired tyrosinase activity. As a copper‐dependent metalloenzyme predominantly expressed in melanocytes, tyrosinase plays a crucial role in melanin biosynthesis. Tyrosinase mainly controls the first two rate‐limiting steps in the melanin biosynthetic pathway: 1. the oxidation of tyrosine to L‐dihydroxy‐phenylalanine (DOPA); 2. the subsequent dehydrogenation of DOPA to dopaquinone [5]. When tyrosinase activity is partially or entirely compromised, melanin synthesis in the skin, hair, and eyes is reduced or absent, resulting in a lifelong phenotypic manifestation. Owing to a total absence of melanin, patients with OCA1 present with nystagmus, strabismus, photophobia, and vision loss, which are severe enough to cause blindness [6]. Despite its high prevalence and severe clinical symptoms, there is no targeted treatment or cure available for OCA1. Supportive care and symptom relief, such as correcting ametropia, preventing sunburn, and low vision assistance, have limited effects on preventing the development of this disease. For diseases with defects in a single gene, gene replacement therapy is the most direct method. Owing to the success of Luxturna, recombinant adeno‐associated virus (rAAV)‐based gene replacement therapies have shown enormous potential in treating inherited retinal diseases. Although it has advantages such as high safety and low immunogenicity [7, 8, 9, 10], transgene expression in unwanted cells may cause toxicity, such as hepatotoxicity [11], neurotoxicity, and even death in critical cases [12, 13]. Gargiulo et al. [14]. developed an AAV2/1 vector carrying the human *TYR* gene, driven by the cytomegalovirus (CMV) promoter, to treat tyrosinase‐deficient albino mice. Their findings revealed that AAV‐mediated delivery of the human *Tyrosinase* gene into the retina could stimulate melanosome formation and enhance melanin production.

Melanocytes in the eyes are present in the choroid, iris stroma, and retinal pigment epithelium (RPE). The RPE is a highly differentiated monolayer of pigment cells derived from the neuroectoderm. The RPE is situated between photoreceptor outer segments and the choroid, serving multiple vital roles in maintaining retinal homeostasis [15, 16, 17, 18, 19, 20]. Its key functions include: (1) phagocytosing shed photoreceptor outer segment discs; (2) absorbing scattered light; (3) modulating immune responses; (4) facilitating nutrient transport to photoreceptors; (5) producing essential growth factors; and (6) storing and metabolizing retinoids. Multiple investigations indicate that melanin in the RPE serves as a crucial photoprotective compound, demonstrating multiple protective functions including free radical neutralization [21], metal ion chelation [22], inhibition of lipid peroxidation [18], and ocular oxidative stress reduction [23]. Additionally, RPE melanin can protect against retinal degeneration [24] and lipofuscin accumulation in the eye [25] and may be an important protective factor against macular dystrophy [21]. Therefore, we hypothesize that RPE‐specific restoration of functional tyrosinase is sufficient to rescue visual function and ameliorate structural abnormalities of the retina in OCA1 patients.

For treating IRDs, rAAVs are delivered mainly through subretinal injections since they can directly deliver vectors to RPE cells and photoreceptor cells, which is usually the preferred method for treating these two types of cell‐related diseases. However, this method involves the injection of vectors into the subretinal space between photoreceptor cells and the RPE, which requires separation of the retinal neuroepithelial layer (RNL) from the RPE. This may cause retinal detachment, glial cell proliferation, photoreceptor degeneration, and visual impairment. Following subretinal vector administration, transfection is predominantly localized to the bleb area—the transient space created between photoreceptors and RPE by the injected fluid. This limited distribution may constrain therapeutic efficacy [26, 27]. Recent advances have identified suprachoroidal injection as a promising alternative approach for ocular drug delivery. The suprachoroidal space, a naturally occurring potential area between the sclera and choroid, can be mechanically expanded through fluid injection. Unlike subretinal delivery, vectors administered via the suprachoroidal route demonstrate circumferential dispersion around the ocular globe, enabling extensive tissue exposure. Studies [26] have revealed that suprachoroidal AAV8‐mediated gene transfer achieves robust transgene expression across RPE and photoreceptor layers in multiple species, including rats, nonhuman primates, and porcine models. This delivery method shows potential for developing minimally invasive, clinic‐based procedures for retinal disorder management. The broader distribution pattern suggests suprachoroidal injection may represent a superior vector delivery strategy for inherited retinal dystrophies compared with conventional subretinal approaches.

In this study, we conducted codon optimization in vitro to increase the expression level of tyrosinase. To validate the therapeutic potential of the RPE‐specific restoration of tyrosinase, we generated AAV8 vectors harboring the optimized human *TYR* gene (hTYRco) under the transcriptional control of the ubiquitous CMV promoter or RPE‐specific promoter (BEST1/RPE65). We delivered all types of vectors to B6 albino mice via subretinal injection or to WISTAR rats, an OCA1 rat model with the same common clinical mutation (p.Arg299His) [28] via subretinal injection or suprachoroidal injection. The results revealed that subretinal administration of the RPE65 promoter‐driven vector effectively reestablished tyrosinase production, recovered both retinal and visual capabilities, and prevented photoreceptor degeneration in OCA1 murine models. Furthermore, compared with traditional subretinal delivery, suprachoroidal delivery retained more visual function and caused a wider range of pigment deposition, indicating that it was more suitable for RPE‐specific gene therapy for OCA1.

## 2 Results

### 2.1 Gene Therapy Restores the Expression of Tyrosinase and Melanin Deposition in the RPE Cells of Albino Mice

The protein expression levels of tyrosinase were initially compared between the wild‐type (hTYRwt) and codon‐optimized human TYR (hTYRco) variants in HEK293 cells (Figure S1). After 72 h of transfection, the cell pellets were collected, and the average optical density value was measured. The results showed that hTYRco significantly improved the biosynthesis of melanin (Figure S1A,B). Tyrosinase protein levels were measured using western blotting. The results revealed that hTYRco significantly increased the protein expression level, which was approximately 1.76‐fold greater than that of hTYRwt (Figure S1C,D).

**FIGURE 1:**
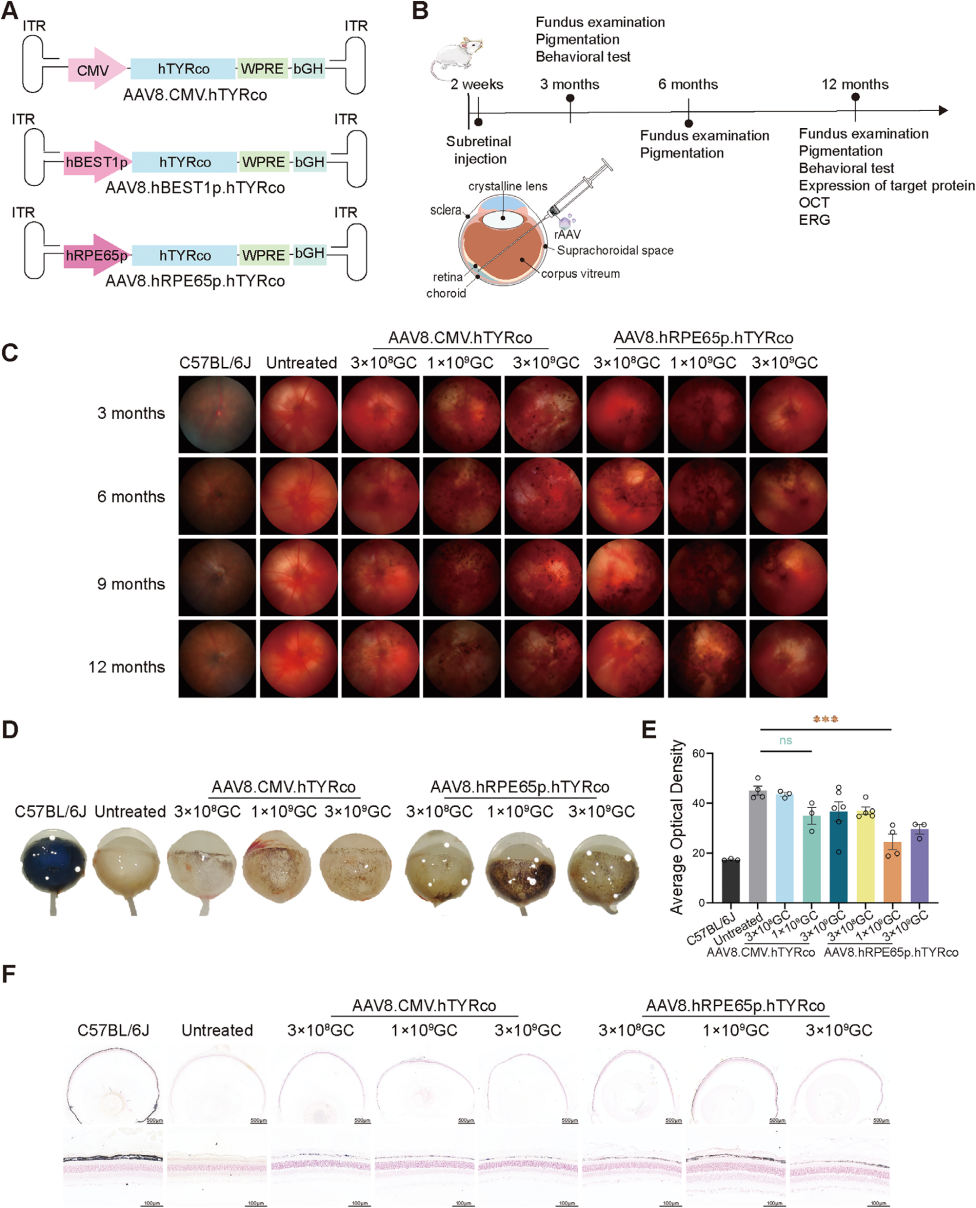
**Subretinal delivery of AAV8.hTYRco restored the expression of *TYR* and melanin deposition. (A)** Schematic of the AAV8.CMV.hTYRco, AAV8.hBEST1p.hTYRco, and AAV8.hRPE65p.hTYRco vectors. ITR: inverted terminal repeats from AAV2; CMV: Cytomegalovirus; hBEST1p: human BEST1 promoter; hRPE65p: human RPE65 promoter; hTYRco: codon optimized human *TYR* gene; bGH: bGH polyadenylation signal. WPRE: woodchuck hepatitis virus posttranscriptional regulatory element. **(B)** Experimental schedule for B6 albino mice. **(C)** Representative fundus images. **(D)** Representative eye images of the mice at 12 months postinjection. **(E)** Analysis of the average optical density of the eye images of the mice at 12 months postinjection (*n* = 3–6 in each group). The data are shown as the mean ± SEM. One‐way ANOVA and post hoc Dunnett's test were used for comparisons with the untreated group. *p < 0.05, **p < 0.01, ***p < 0.001. **(F)** Display of pigment deposition in the RPE layer through Masson‒Fontana staining.

To encode the full‐length hTYRco protein, we selected a ubiquitous promoter, CMV, and two human RPE cell‐specific promoters, BEST1 and RPE65, to construct three candidate vectors, namely, AAV8.CMV.hTYRco, AAV8.hBEST1p.hTYRco, and AAV8.hRPE65p.hTYRco (Figure 1A).

Two‐week‐old albino mice underwent bilateral subretinal injections (Figure 1B). Each eye received 1 µL of AAV8 vector containing either CMV, hBEST1, or hRPE65 promoter‐driven hTYRco (AAV8.CMV.hTYRco, AAV8.hBEST1p.hTYRco, or AAV8.hRPE65p.hTYRco) at three different concentrations: low (3 × 10⁸ GC/eye), medium (1 × 10⁹ GC/eye), or high (3 × 10⁹ GC/eye) doses. To evaluate the efficacy of different vectors at specific dosages, ocular melanin deposition was detected at 3 months postinjection (Figure S2A). The results demonstrated that all three vectors successfully induced melanin production. At the same dose, the AAV8.hRPE65p.hTYRco treatment group presented the greatest melanogenesis, with a significantly greater melanogenesis rate in the medium‐dose group than in the untreated group. The groups treated with AAV8.hBEST1p.hTYRco presented the least melanogenesis, and there was no significant difference between all the AAV8.hBEST1p.hTYRco groups and the untreated group (Figure S2B). Therefore, AAV8.hRPE65p.hTYRco and AAV8.CMV.hTYRco were used to evaluate the long‐term efficacy of RPE‐specific *TYR* supplementation. At 12 months postinjection, tyrosinase expression was evaluated via western blot analysis. The results revealed that tyrosinase expression in the AAV8.CMV.hTYRco treatment group of mice was greater than that in the AAV8.hRPE65p.hTYRco group at the same dose, and both groups presented the highest protein expression at the medium dose, which was 2.28‐fold (AAV8.CMV.hTYRco) and 1.15‐fold (AAV8.hRPE65p.hTYRco) greater than that of C57BL/6J (Figure S3A,B). We also noted that untreated mice expressed a small amount of tyrosinase (Figure S3). To confirm that tyrosinase in OCA1 mice is nonfunctional, we generated a plasmid expressing full‐length *mTYR* mRNA carrying the p.Arg77Leu mutation (musTYRmut) and transfected it into HEK293 cells. After 72 h of transfection, the cell pellets were collected, and the results revealed that the transfection of the normal *TYR* gene (musTYRwt) promoted melanin biosynthesis in HEK293 cells, whereas musTYRmut did not (Figure S4A). Immunofluorescence was performed on the collected cells. The results revealed that cells transfected with musTYRwt or musTYRmut were positive for tyrosinase (Figure S4B). These data indicated that tyrosinase in untreated mice is nonfunctional. Fundus photography revealed a significant accumulation of melanin with time after treatment (Figure 1C). To quantitatively assess the deposition of melanin, we measured the average optical density of mice's eyeballs. The results revealed that the hRPE65 promoter was more effective than the CMV promoter, increasing the production of melanin and significantly reducing the translucency of treated mouse eyeballs (Figure 1D,E).

The eyeballs were fixed and embedded in paraffin, after which Masson‐Fontana staining was performed. The results revealed that the entire eyeballs of the treated mice were almost uniformly stained with melanin, with the mice treated with medium‐dose AAV8.hRPE65p.hTYRco exhibiting the most pigmentation (Figure 1F). To understand the reasons for the decrease in protein expression and pigmentation at high doses, we measured the expression levels of inflammatory factors in all dose groups of the AAV8.hRPE65p.hTYRco. Compared with those in the noninjection group and the low‐dose and medium‐dose groups, the expression levels of TNFα, IL‐1β, IL‐6, and IFN‐γ in the eyes of the high‐dose group were significantly greater (Figure S5). Based on previous research [29], we speculated that this may be caused by the overexpression of tyrosinase or high‐dose AAV toxicity leading to partial cell death.

### 2.2 Vector with Specific Promoter Improves Retinal and Visual Function in B6 Albino Mice to a Greater Extent

Previous studies have reported functional retinal abnormalities in albino mice due to melanin deficiency and progressive photoreceptor degeneration caused by ambient light. As early as 1 month of age, a modest but significant reduction in the electroretinography (ERG) response can be detected at high luminance intensities, which further decreases at 6 months of age [14]. Similar progressive degeneration of visual function in B6 albino mice was observed in this study (Figure S6).

To assess whether partial recovery of melanin synthesis could enhance photoreceptor activity, a full‐field ERG was recorded in mice. This electrophysiological technique allows simultaneous measurement of both rod‐dominated scotopic and cone‐mediated photopic visual responses.

**FIGURE 2:**
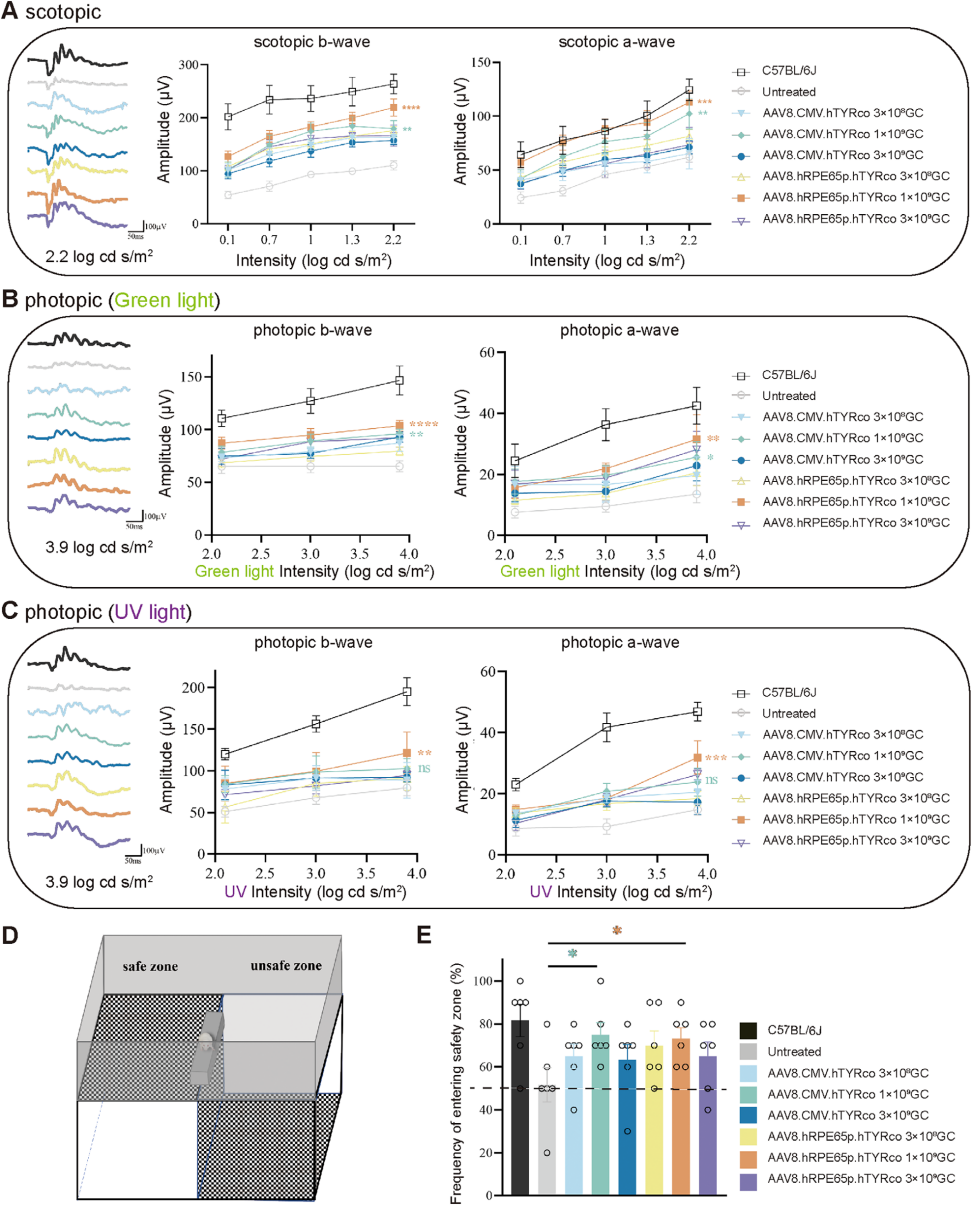
**Restoration of retinal and visual function in B6 albino mice after gene therapy. A, B, C** Representative ERG waveforms (left), b‐ and a‐wave amplitudes at 12 months postinjection (middle two) under scotopic **(A)**, green light photopic **(B),** and UV light photopic **(C)** conditions. The representative ERG waveforms on the left are recorded under stimulation with a light stimulus of 2.2 log cd s/m^2^ in scotopic and 3.9 log cd s/m^2^ in photopic conditions. Two‐way ANOVA and post hoc Dunnett's test were used for comparisons with the untreated group, as shown in the middle two graphs. *p < 0.05, **p < 0.01, ***p < 0.001, ****p < 0.0001; ns, nonsignificant difference. **(D)** Visual cliff test assembly composed of a safe zone and an unsafe zone. The number of times the mice selected the safe zone was recorded. **(E)** Quantitative analysis of 10 step‐down choices of mice 12 months postinjection. The black dotted lines indicate no preference for safe zone or unsafe zone, with 50% of the choices each. *n* = 6 for each group. The data are shown as mean ± SEM. One‐way ANOVA and post hoc Dunnett's test were used for comparisons with the untreated group. *p < 0.05, **p < 0.01, ***p < 0.001; ns, nonsignificant difference.

Scotopic and photopic ERG analysis demonstrated that, at 12 months postinjection, eyes treated with either AAV8.CMV.hTYRco or AAV8.hRPE65p.hTYRco exhibited significant increases in b‐wave and a‐wave amplitudes under scotopic and green light conditions at higher stimulus intensities compared with untreated controls. This enhancement was observed across all three administered doses. Notably, mice receiving the medium dose of AAV8.hRPE65p.hTYRco displayed more pronounced improvements than those treated with the equivalent dose of AAV8.CMV.hTYRco. At the maximal stimulation, the b‐ and a‐wave amplitudes showed significant enhancement. Specifically, scotopic ERG recordings demonstrated increases of 22.4% (b‐wave) and 10.2% (a‐wave) (Figure 2A). Photopic ERG responses exhibited differential improvements: green light stimulation resulted in 7.8% (b‐wave) and 26.7% (a‐wave) augmentation (Figure 2B), while UV light exposure produced more pronounced gains of 18.2% (b‐wave) and 33.8% (a‐wave) (Figure 2C).

To demonstrate that heightened rod and cone responses enhance visual function in mice, we conducted an adapted visual cliff assay to evaluate visually guided behavior. During the test, individual mice were positioned on a central platform and given the freedom to move toward either the secure or hazardous zone (Figure 2D). In the 10 step‐down trials, the mice treated with a medium dose of AAV8.hRPE65p.hTYRco or AAV8.CMV.hTYRco presented significantly better performance, with a greater frequency in the safe zone (hRPE65: 7.0 ± 0.5 and CMV: 7.0 ± 0.3) than did the untreated mice (5.0 ± 0.2) (Figure 2E). The results of the step‐down trials were consistent with the ERG results, indicating that in vivo gene therapy improved retinal and visual function in B6 albino mice.

### 2.3 Vector with Specific Promoter Can More Effectively Prevent Photoreceptor and Outer Nuclear Layer Loss

**FIGURE 3:**
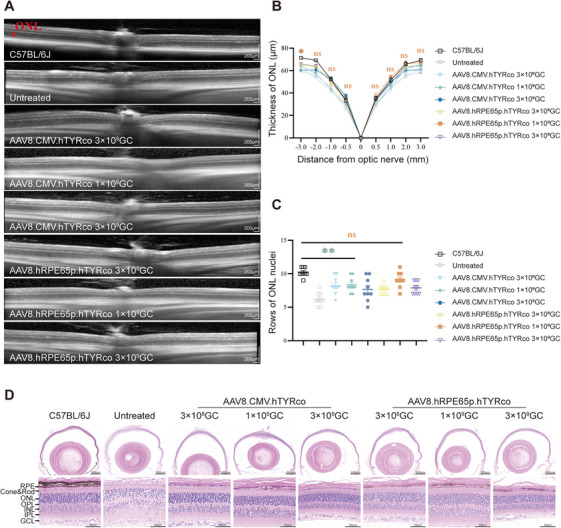
**Retinal structures are rescued in treated B6 albino mice after gene therapy. (A)** Representative OCT images from 12‐month‐old WT mice and untreated and treated B6 albino mice. The red bar represents the ONL layer. **(B)** The results of ONL thickness measurements at different distances from the optic disc (*n* = 8 for each group). The data are shown as the mean ± SEM. Two‐way ANOVA and post hoc Dunnett's test were used for comparisons with the C57BL/6J group. *p < 0.05; ns, nonsignificant difference. **(C)** Results of the average rows of ONL nuclei at three different positions on the cut surface of H&$E staini (n = 3 for each group). The data are shown as the mean ± SEM. One‐way ANOVA and post hoc Dunnett's test were used for comparisons with the C57BL/6J group. *p < 0.05, **p < 0.01; ns, nonsignificant difference. **(D)** Representative H&E images of mice 12 months postinjection

Progressive degeneration of photoreceptors and apoptosis of the outer nuclear layer (ONL) caused by environmental light are among the main causes of visual impairment in albino mice. To determine whether the recovery of melanin deposition could effectively block light damage and prevent photoreceptor loss, we evaluated the structure of the retina via optical coherence tomography (OCT) and hematoxylin and eosin (H&E) staining at 12 months postinjection. OCT was used to measure the ONL thickness at various positions around the optic disc. Quantitative assessments showed that the ONL thickness ranged from 34.9 to 69.0 µm in wild‐type (WT) mice and 28.9 to 58.5 µm in untreated mice. The thicknesses of the ONL layer in treated mice were generally greater than those in untreated mice, with the medium‐dose group of AAV8.hRPE65p.hTYRco having the thickest ONL layer at 36.6–67.0 µm, comparable to that of WT mice (Figure 3A,B). Histological analysis of the retinas of 12‐month‐old mice revealed similar results. A significant reduction in rows of nuclei in the ONL layer was observed in untreated mice (∼7 rows of nuclei), whereas the ONL layer of mice treated with a medium dose of AAV8.hRPE65p.hTYRco (∼9 rows of nuclei) was well protected compared with that of WT mice (∼10 rows of nuclei) (Figure 3C,D). In addition, we also observed that the nuclear distribution in untreated mice was relatively loose, whereas the nuclear distributions in wild‐type mice and treated mice were more compact.

Collectively, these results indicate that gene therapy delivered through AAV8 could restore tyrosinase expression and melanin deposition in the RPE of B6 albino mice, thereby protecting the retina from environmental light damage, preventing photoreceptor loss, and sustaining normal retinal structure and retinal and visual function. In addition, the results suggested that AAV8.hRPE65p.hTYRco was more efficient than AAV8.CMV.hTYRco and reached its optimum at medium doses.

### 2.4 Suprachoroidal Delivery of Vectors Results in Wider Distribution

Although we demonstrated that subretinal delivery of AAV8.hRPE65p.hTYRco restored the visual function of B6 albino mice, several limitations, including a limited area of AAV infection and a high incidence of retinal detachment or atrophy, may influence the outcome. Therefore, finding a safer and more efficient way for AAVs to be delivered to the RPE is highly important. Suprachoroidal injection has recently been demonstrated to provide a new route for ocular drug delivery [30, 31]. This method delivers AAVs to the potential space on the inner surface of the sclera, which can expand the area of AAV infection and may be more suitable for treating OCA1 [32, 33].

**FIGURE 4:**
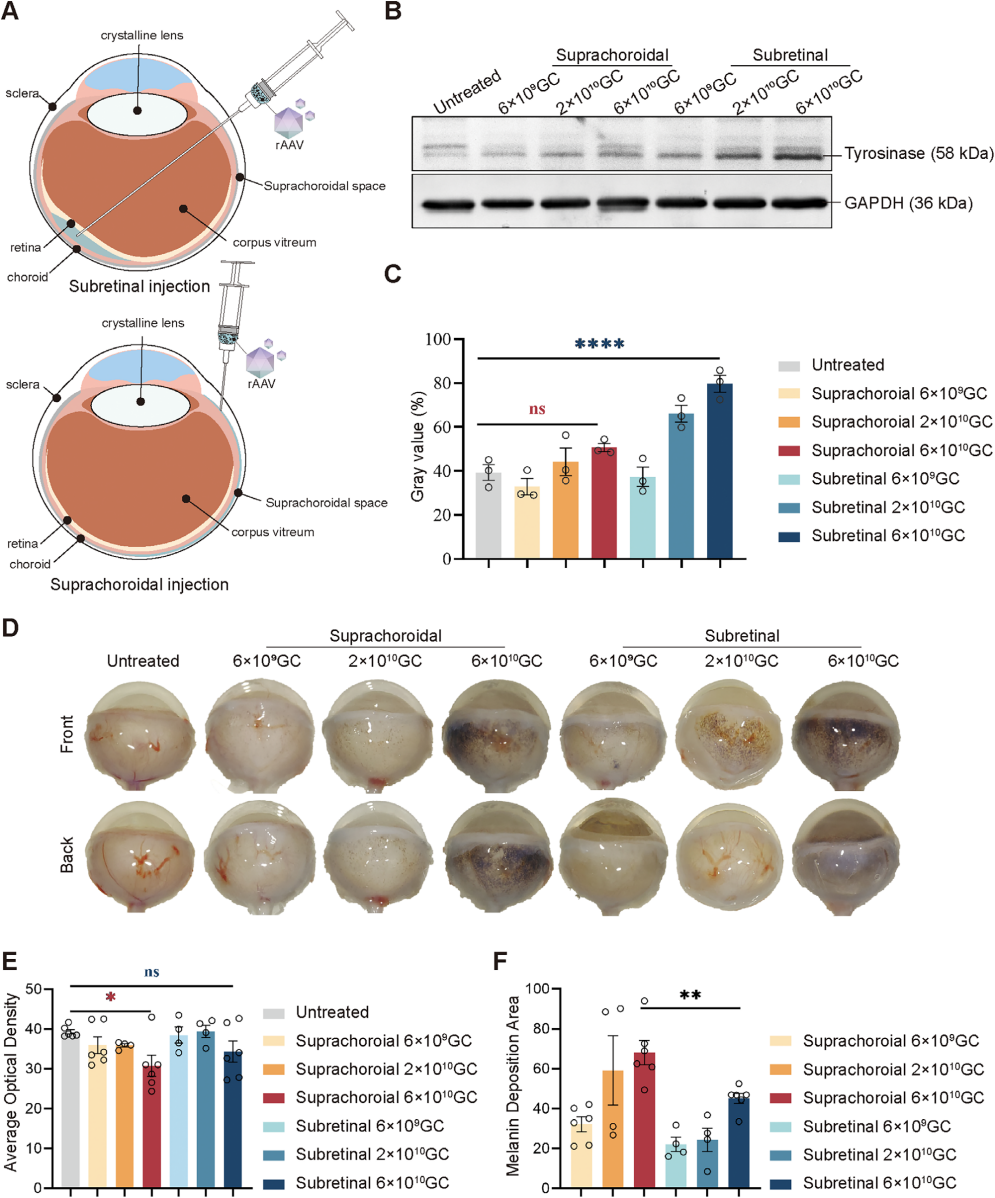
**Subretinal and suprachoroidal cavity space delivery of AAV8.hRPE65p.hTYRco restored the expression of *TYR* and melanin deposition. (A)** Schematic of subretinal (top) and suprachoroidal cavity space (bottom) delivery. **(B)** Representative western blot analysis of untreated and treated WISTAR rat retinas at 2 months postinjection. GAPDH (36 kDa) was used as a loading control. **(C)** Gray value analysis of the western blot in **(B)**. The data are shown as the mean ± SEM. One‐way ANOVA and post hoc Dunnett's test were used for comparisons with the untreated group. *p < 0.05, **p < 0.01, ***p < 0.001, ****p < 0.0001; ns, nonsignificant difference. **(D)** Representative images of the front and back of rat eyes at 12 months postinjection. The top and bottom represent the front and back of the same eye, respectively. **(E)** Analysis of the average optical density from eye images of rats at 12 months postinjection (*n* = 4–6 in each group). The data are shown as the mean ± SEM. One‐way ANOVA and post hoc Dunnett's test were used for comparisons with the untreated group. *p < 0.05; ns, nonsignificant difference. **(F)** Quantitative analysis of the melanin coverage area in rat eyeballs (*n* = 4–6 in each group). The data are shown as the mean ± SEM. *p < 0.05 (unpaired *t*‐est).

To confirm whether injection into the suprachoroidal cavity space can increase the area of AAV infection, we compared the two injection methods: suprachoroidal cavity space delivery and subretinal delivery in WISTAR rats (Figure 4A). Two‐week‐old WISTAR rats received either suprachoroidal or subretinal injections. Both eyes of each animal were administered 5 µL of AAV8 (AAV8.hRPE65p.hTYRco) at three different concentrations: low (6 × 10^9^ GC/eye), medium (2 × 10^10^ GC/eye), and high (6 × 10^10^ GC/eye) doses. Eye samples were taken from euthanized rats at 3 months postinjection, and tyrosinase expression was evaluated via western blotting (Figure 4B). The results revealed that more tyrosinase was expressed in the rats subjected to subretinal delivery, and the expression level of tyrosinase increased with increasing dose (Figure 4C). In addition, WISTAR rats, like B6 albino mice, presented corresponding target bands. We conducted in vitro validation similar to that of the mice mentioned earlier in this study. The results revealed that HEK293 cells transfected with plasmids expressing full‐length WISTAR rat *TYR* mRNA, which carried the p.Arg299His mutation, did not accumulate melanin (Figure S7A), but immunofluorescence revealed positive tyrosinase expression (Figure S7B), confirming that WISTAR rats expressed nonfunctional tyrosinase. Twelve months after delivery, pigmentation was evident in the enucleated eyes in the portion of the retina exposed to the vector (Figure 4D). The average optical density of the rat eyeballs was measured to evaluate melanin deposition. The results showed that at the high dose, the average optical density of rat eyeballs delivered through the suprachoroidal cavity space was significantly lower than that of untreated rats, indicating a significant decrease in light transmittance. There was no significant difference observed in rats that delivered via the subretina (Figure 4E). Statistics on the pigmented area of rat eyeballs revealed that the pigmented area increased with increasing dose. At the high dose, the pigmented area of the rats delivered via the suprachoroidal cavity reached 68.14%, which was 1.5‐fold greater than that of the rats delivered via the subretinal cavity (Figure 4F). The results clearly showed that subretinal delivery induced strong but localized melanin deposition, whereas suprachoroidal injection resulted in weaker but widespread melanin deposition, indicating a larger infectious area of AAV.

### 2.5 A Wider Distribution of AAV8 Improves the Visual Function of WISTAR Rats More Effectively

**FIGURE 5:**
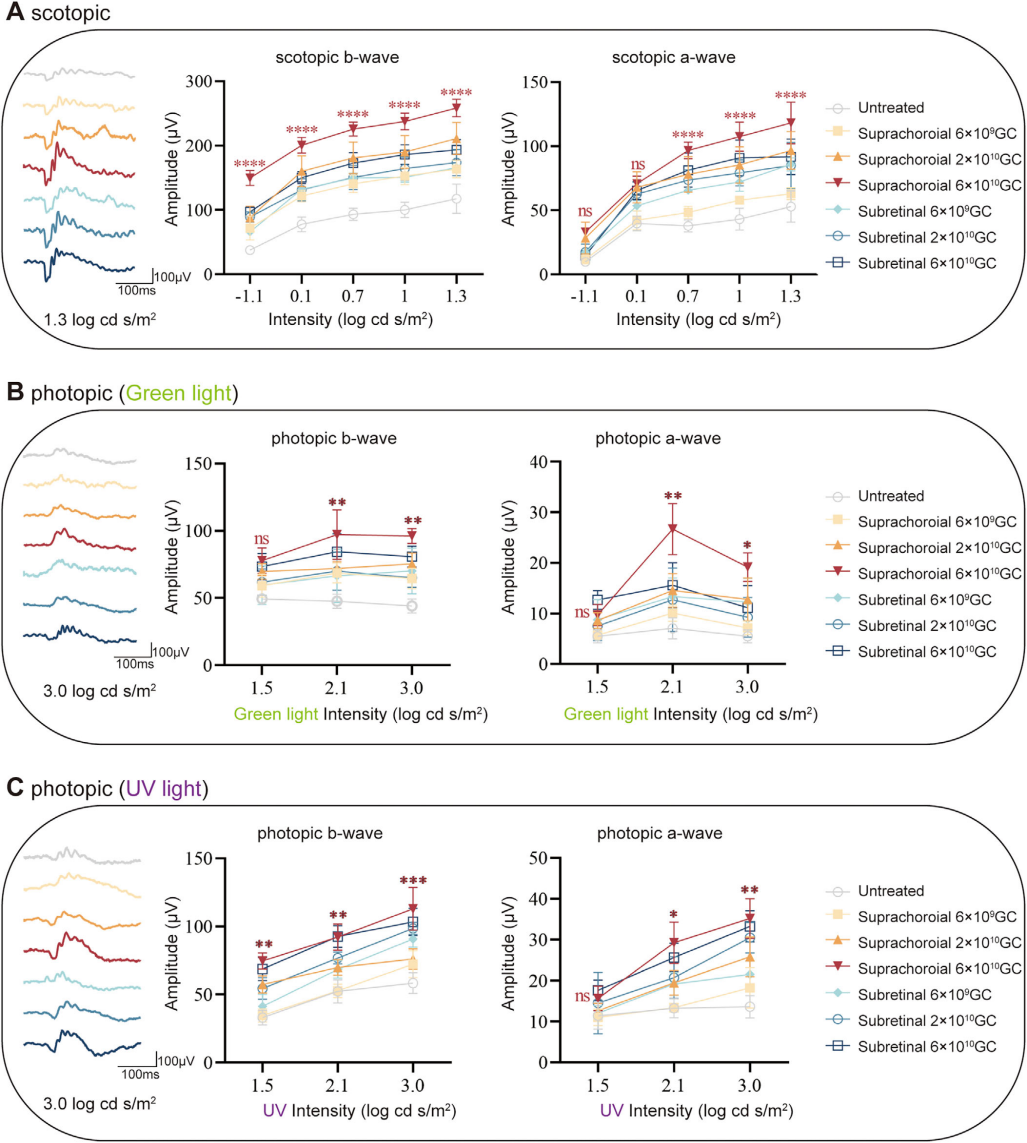
**Restoration of retinal function in WISTAR rats after gene therapy. (A, B, C)** Representative ERG waveforms (left) and b‐ and a‐wave amplitudes at 12 months postinjection (middle two) under scotopic **(A)**, green light photopic **(B)**, and UV light photopic **(C)** conditions. The representative ERG waveforms on the left are recorded under stimulation with a light stimulus of 1.3 log cd s/m^2^ in scotopic and 3.0 log cd s/m^2^ in photopic conditions. Two‐way ANOVA and post hoc Dunnett's test were used for comparisons with the untreated group, as shown in the middle two graphs. *p < 0.05, **p < 0.01, ***p < 0.001, ****p < 0.0001; ns, nonsignificant difference.

To determine whether the expansion of melanin deposition can further salvage the function of rods and cones in the long term, full‐field ERG has been performed in rats. The scotopic and photopic ERG revealed that at 12 months postinjection, compared with untreated rats, those treated with medium and high doses presented increased amplitudes of b‐ and a‐waves at relatively high stimulation intensities. Compared with those that received subretinal delivery, rats that received high‐dose suprachoroidal delivery presented more prominent increases in the amplitudes of b‐ and a‐waves. Under maximal stimulation, the b‐ and a‐wave amplitudes exhibited significant enhancements across different ERG conditions, 33.6% and 28.7% increase, respectively, in scotopic ERG (Figure 5A); 19.0% and 72.0% improvement, respectively, in green light photopic ERG (Figure 5B); and 9.3% and 5.8% augmentation, respectively, in UV light photopic ERG (Figure 5C). The results confirmed that both delivery methods protected the rats from loss of visual function in the long term. More importantly, the data suggested that modest but widespread melanin production induced by suprachoroidal injection achieved better effects.

### 2.6 Administration Through the Suprachoroidal Cavity Space Is Safer Than That Through the Subretinal Space

**FIGURE 6:**
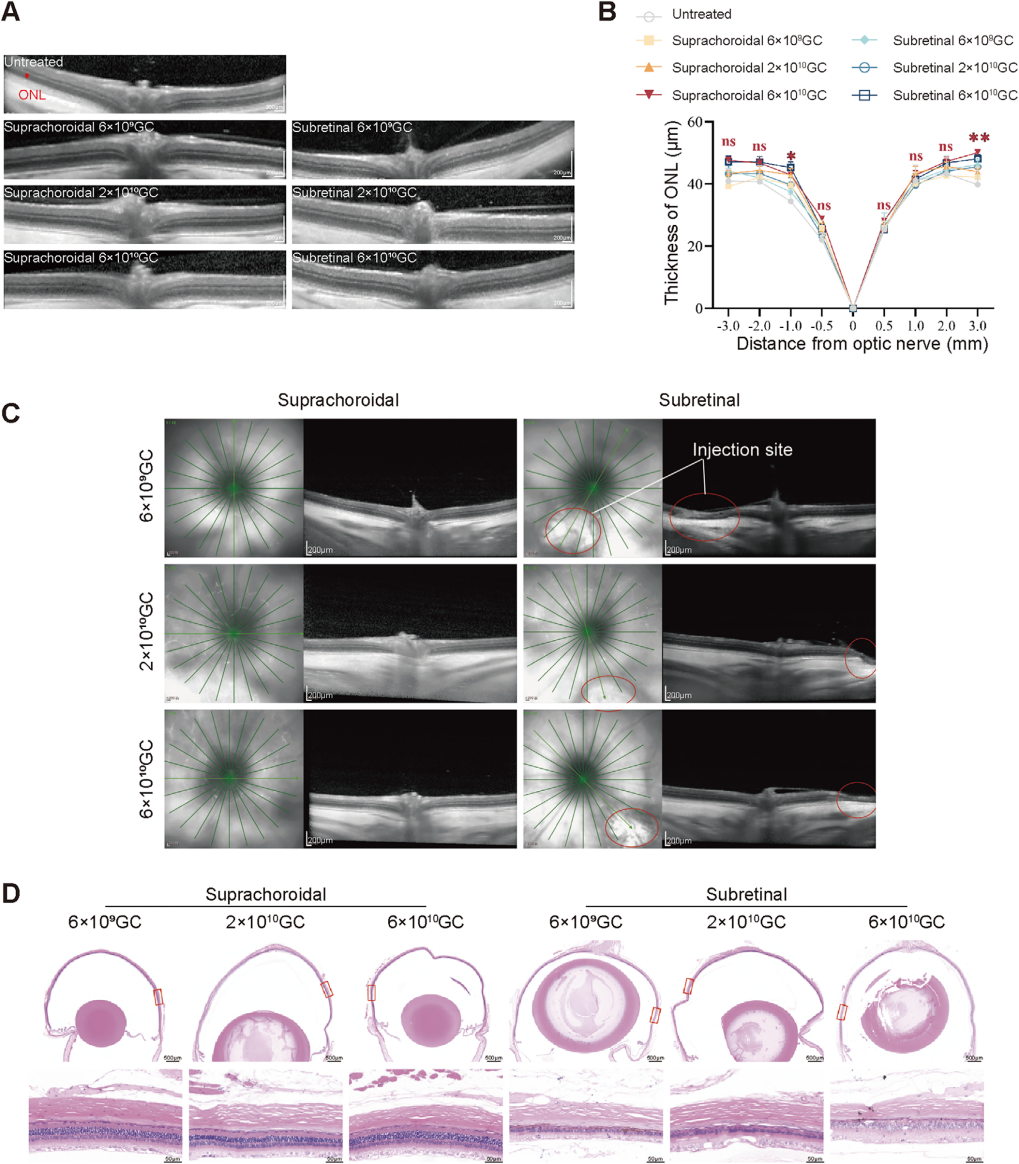
**Administration through the suprachoroidal cavity space is safer than that through the subretinal cavity. (A)** Representative OCT images of untreated and treated WISTAR rats at 12 months postinjection. **(B)** ONL thickness measurements at different distances from the optic disc (*n* = 6 in each group). The data are shown as the mean ± SEM. Two‐way ANOVA and post hoc Dunnett's test were used for comparisons with the untreated group. *p < .05, **p < 0.01; ns, nonsignificant difference. **(C)** Representative OCT images of rats delivered via the suprachoroidal cavity space and subretinal space. The white circle represents the injection site. **(D)** Representative H&E‐stained images of rats delivered via the suprachoroidal cavity space and subretina.

At 12 months of age, OCT detection and analysis of ONL layer thickness were performed on untreated and treated rats. The thickness of the ONL across the optic disc at multiple positions was measured via OCT, and quantitative analysis revealed that the ONL thickness in untreated rats was 25.0–41.3 µm. The ONL layer of the high‐dose treatment group rats was generally thicker than that of the untreated rats, with the ONL layer being thickest in the high‐dose group of rats delivered via the suprachoroidal cavity space at 28.8–50.0 µm (Figure 6A,B).

As mentioned earlier, subretinal delivery may present certain risks, such as retinal detachment and atrophy. We assessed the retinal structure at the injection site in eyes subjected to suprachoroidal or subretinal injection via OCT and H&E staining and found that the retinas of some rats subjected to subretinal injection exhibited regional atrophy at the injection site, but this effect was not observed in any of the eyes of the rats that received suprachoroidal injection (Figure 6C,D). The results indicated that suprachoroidal cavity space injection not only allowed for a wider distribution of AAVs but also prevented regional retinal atrophy caused by subretinal injection. Together, these data highlighted that RPE‐specific gene therapy with AAV8.hRPE65p.hTYRco achieved efficient and long‐term therapeutic effects on OCA1. RPE‐specific gene therapy is feasible for restoring melanin production widely and safely via suprachoroidal cavity injection in rats with albinism.

## 3 Discussion

In this study, we developed an AAV‐mediated gene therapy strategy for the treatment of OCA1. This strategy involves the design of a vector containing an RPE‐specific promoter (RPE65) and a codon‐optimized human TYR gene. Delivering the vector to the eyes of mice or rats at P14 can effectively restore functional tyrosinase expression and melanin biosynthesis in the RPE, retain retinal and visual function, and protect the eyes from light damage. Moreover, we demonstrated that administering vectors through the suprachoroidal cavity space could infect more cells and achieve better therapeutic effects. Moreover, suprachoroidal cavity administration is safer and more convenient. This preclinical study provides a potentially feasible approach for the clinical treatment of OCA1.

Tyrosinase is a key rate‐limiting enzyme in melanin biosynthesis, and its absence can lead to pigment deficiency‐related diseases such as albinism. The overexpression of tyrosinase also has certain cytotoxic effects, leading to increased expression of related inflammatory factors. It has been reported that tyrosinase increases the toxicity of H_2_O_2_ and the oxidizing agent paraquat [34]. H_2_O_2_ exacerbates oxidative stress and suppresses dopamine transporter activity [35]. Meanwhile, the oxidizing compound paraquat stimulates dopamine production by enhancing tyrosine hydroxylase function [36]. Since tyrosinase facilitates the conversion of dopamine into dopamine quinones, the generation of these reactive oxidative derivatives may serve as a primary mechanism underlying heightened toxicity. The cytotoxic effects of dopamine quinones could stem from their interaction with α‐synuclein [34]. Furthermore, Hasegawa et al. [37] demonstrated that elevated tyrosinase expression results in intracellular accumulation of dopamine and reactive oxygen species (ROS), ultimately inducing cellular toxicity. Therefore, supchoroidal injection of vectors with RPE‐specific promoter might be an effective strategy to prevent potential cytotoxicity caused by off‐target effects or local overexpression of tyrosinase.

Mice that received high‐dose treatment presented reduced melanin biosynthesis in the early stages, followed by decreased expression of tyrosinase protein and increased levels of inflammatory factors in the later stages. Although we confirmed that the restoration of tyrosinase can have therapeutic effects, the biosynthesis of melanin is a complex process that is regulated by multiple pathways. OA1 is a melanosome‐localized GPCR that is important for maintaining premelanosome composition [38]. OA1 regulates melanin biosynthesis by modulating the expression level of PMEL17, a melanosomal structural protein that is responsible for fiber formation in early‐stage melanosomes [39]. Previous studies suggested that L‐DOPA, a crucial intermediate in melanin biosynthesis generated by tyrosinase, may serve as the ligand for OA1 [40]. Additionally, OA1 overexpression was found to disrupt the lysosomal trafficking of PMEL17, resulting in the accumulation of PMEL17 within multivesicular endosomes/bodies [41]. In this study, 3 months after injection into mice, tyrosinase expression increased with increasing dose (Figure S8A,B), whereas melanin biosynthesis peaked at a dose of 1 × 10^9^ GC (Figure S8C), which may be related to the aforementioned regulatory mechanism. Additionally, inflammatory mediators are involved in melanogenesis [42]. These chemical mediators modulate melanogenesis primarily by regulating the expression of MITF target genes, including TYR, TYRP1, and DCT, either positively or negatively. Key inhibitory cytokines, such as IL‐1 (α/β), IL‐6, and TNF, suppress melanin production through distinct mechanisms [43]. In detail, IL‐1β downregulates MITF downstream targets via NF‐κB and JNK signaling pathways [44]. IL‐6, which is upregulated by IL‐17, acts synergistically with TNF to further inhibit melanogenesis [45]. Additionally, IFN‐γ—a widely expressed cytokine—reduces melanin synthesis by suppressing TYR, DCT, and TYRP1 expression through both MITF‐dependent and independent pathways [46]. Beyond its direct effects, IFN‐γ also counteracts the promelanogenic activity of other factors, such as 5‐HT and IL‐18 [47, 48]. These underlying mechanisms might account for the reduced tyrosinase expression in the high‐dose treatment group at 12 months after injection (Figures S3 and S5).

Owing to melanin deficiency, excessive light‐induced light transduction signals can lead to experimental retinal degeneration in albino mice. In this study, we showed that melanin synthesis after *TYR* gene complementation protected the albino retina from light‐induced photoreceptor loss. Gargiulo et al. [14] reported that pigmentation in certain areas of the retina can benefit the entire retina of mice. In this study, the percentage of melanin coverage in rats (45%, high dose) injected subretinally was much lower than that in mice, and the benefits shown to the retina were also limited. Furthermore, statistical data indicate that subretinal injections in humans lead to localized infections affecting approximately 10% of retinal or retinal pigment epithelial (RPE) cells. Consequently, the therapeutic efficacy of subretinal delivery for OCA1 treatment may be suboptimal, suggesting that suprachoroidal injection could represent a more viable and efficient alternative.

In summary, the results of this study showed that an appropriate dosage of AAVs could promote sufficient biosynthesis of melanin, which reversed defects caused by pigment deficiency in albino murine models after birth. However, owing to limitations in disease models, we cannot confirm whether the recovery of melanin biosynthesis after birth can also reverse the abnormal retinal development caused by pigment deficiency during embryonic development, such as central foveal hypoplasia. In addition, this study indirectly reflected the possible complex regulation between the biosynthesis of melanin and the expression of tyrosinase, suggesting that in gene therapy for similar genetic diseases, it is necessary not only to pay attention to the expression of supplemented defective genes but also to explore the related proteins and upstream and downstream pathways in depth.

## 4 Materials and Methods

### 4.1 Codon Optimization

In this study, codon optimization was performed using the online GenSmart Codon Optimization tool. The optimization process carefully balanced multiple factors, including GC content, codon usage frequency, RNA splicing sites, and RNA‐stabilizing trans‐acting elements, ensuring appropriate weighting of all critical parameters.

### 4.2 Plasmid Construction

The *TYR* genes of wild‐type and codon‐optimized human, B6 albino mouse models, and WISTAR rats were synthesized by Genewiz (Suzhou, China). We constructed the AAV vector for in vitro validation, containing the codon‐optimized human (wild‐type human/ mouse/ rat) *TYR* gene, the CMV promoter, and bGH polyadenylation sequence, yielding the pAAV.hTYRco, (pAAV.hTYRwt/ pAAV.musTYRmut/ pAAV.ratTYRmut) plasmid vector. The plasmids pAAV.musTYRwt and pAAV.ratTYRwt were obtained by mutating a single base of the *TYR* gene of the plasmids pAAV.musTYRmut and pAAV.ratTYRmut, respectively. The plasmid vector pAAV.CMV.hTYRco was obtained by adding the WRPE sequence to the plasmid vector pAAV.hTYRco. The pAAV.hBEST1p.hTYRco and pAAV.hRPE65p.hTYRco plasmids were constructed by replacing the CMV of pAAV.CMV.hTYRco with the promoters hBEST1 and hRPE65. All the constructed plasmids were verified by sequencing.

### 4.3 Cell Culture and Transfection

Human embryonic kidney 293 (HEK293) cells, acquired from ATCC (Manassas, USA), were maintained in Dulbecco's modified Eagle medium (DMEM) (Gibco, Carlsbad, USA) supplemented with 10% fetal bovine serum (FBS) (PAN Biotech) and 1% penicillin/streptomycin (100 U/mL) (Boster Biological Technology Co. Ltd.) at 37°C under 5% CO_2_. Before transfection, cells were seeded in 12‐well plates at a density of 7 × 10⁵ cells per well and allowed to adhere for 24 h. The plasmids pAAV.TYRco and pAAV.TYRwt were transfected at 1 µg per well. After 72 h, cells were collected for western blot analysis.

### 4.4 Western Blot

The cells were washed with ice‐cold PBS and lysed in 50 µL RIPA buffer (Beyotime) containing 1% protease inhibitors (Sigma) at 4°C for 30 min with vortexing every 15 min to produce protein from the cell lysates. The eye (without lens) was washed with ice‐cold PBS and added to 50 µL of ice‐cold RIPA buffer with 1% protease inhibitors before sterile scissors were used to cut the samples, which were subsequently incubated at 4°C for 1 h with vortexing every 15 min to prepare the protein from the eye of the mouse. The protein supernatant was isolated by centrifuging the lysate at 17,000×*g* for 15 min at 4°C. Total protein concentration was determined using a BCA protein assay (Thermo Scientific). For immunoblotting, 20 µg of protein from cell lysates or ocular tissues was resolved by SDS‐PAGE and electrotransferred onto PVDF membranes. After blocking with 5% skim milk in TBST for 2 h at room temperature, the membranes were probed with primary antibodies overnight at 4°C, including mouse anti‐tyrosinase monoclonal antibody (1:1000; Santa Cruz) and mouse anti‐GAPDH polyclonal antibody (ABclonal). Following TBST washes, membranes were incubated with HRP‐conjugated goat anti‐mouse IgG secondary antibody (1:10,000; Zsbio) for 1 h at room temperature. Protein signals were detected using an iBright CL1000 imaging system (Thermo Scientific).

### 4.5 AAV Vector Production

The AAV8.CMV.hTYRco, AAV8.hBEST1p.hTYRco, and AAV8.hRPE65.hTYRco vectors were obtained by packaging the pAAV.CMV.hTYRco, pAAV.hBEST1p.hTYRco, and pAAV.hRPE65p.hTYRco plasmids into AAV8, respectively. All AAV8 vectors were produced by packaging the genes by triple plasmid transfection of human embryonic kidney 293 cells as previously described [49]. HEK293 cells (3 × 10⁸) were seeded into 10‐layer cell stacks (Corning, NY, USA) and transfected with PEI‐Max at 75%–90% confluency. A plasmid ratio of 2:1:1 was used, with PEI‐Max to DNA at a 2:1 (w/w) ratio. The plasmids and PEI‐Max were mixed, vortexed, and incubated at room temperature for 15 min before dilution in 1 L of serum‐free DMEM (SFM). The transfection mixture was then replaced with the existing medium in the cell stacks. Cells were maintained at 37°C with 5% CO_2_, and an additional 500 mL of SFM was supplemented at 72 h posttransfection. After another 48 h of incubation, the virus was harvested. Purification was carried out by gradient ultracentrifugation with iodixanol. The genomic titers (GC/mL) of the AAV8 vectors were measured using digital droplet polymerase chain reaction (ddPCR) with the forward primer 5′ TAGTTGCCAGCCATCGTTG‐3′, reverse primer 5′‐TAGGAAAGGACAGTGGGAGT‐3′, and probe 5′‐Fam‐CCCGTGCCTTCCTTGACCCT‐BHQ‐3′. All vectors used in this study passed the endotoxin assay via the QCL‐1000 Chromogenic LAL Test Kit (Cambrex, East Rutherford, NJ, USA).

### 4.6 Animals

B6(Cg)‐Tyr^c‐2J/J^ (B6 albino) mice were sourced from the Jackson Laboratory (Bar Harbor, USA). Wild‐type (WT) C57BL/6J mice were procured from Chengdu Dossy Experimental Animals Co., Ltd. (China), while WISTAR rats were obtained from Beijing Vital River Laboratory Animal Technology Co., Ltd. (China). The animals were kept in a light‐controlled room and had unrestricted access to food and water during a 12 h light–dark cycle. Unless otherwise indicated, all experimental procedures involving mice and rats were performed under anesthesia induced by intraperitoneal injection of xylazine (12 mg/kg) and ketamine (80 mg/kg). Before ocular manipulations, pupil dilation was achieved using a topical solution containing 0.5% tropicamide and 0.5% phenylephrine hydrochloride.

### 4.7 Subretinal Injection

Subretinal injections were performed in two‐week‐old mice anesthetized with isoflurane. Following pupillary dilation, a corneal limbal incision was created using a 31‐gauge needle under stereomicroscopic guidance. A blunt 33‐gauge Hamilton needle was then carefully inserted through this incision to access the subretinal space while avoiding lens injury. Each eye received a volume with 1 µL of three doses of AAV8 (AAV8.CMV.hTYRco, AAV8.hBEST1p.hTYRco, and AAV8.hRPE65p.hTYRco) (3 × 10^8^ GC/eye, 1 × 10^9^ GC/eye, and 3 × 10^9^ GC/eye). Each eye of each rat was injected with 5 µL of AAV8.hRPE65p.hTYRco in three doses (6 × 10^9^ GC/eye, 2 × 10^10^ GC/eye, and 6 × 10^10^ GC/eye). Following injection, the fundus was immediately examined using a fundus imaging microscope (Micron IV, Phoenix Research Labs). Successful subretinal injections were defined as those producing subretinal blebs without substantial subretinal hemorrhage or vitreous bleeding. After the procedure, ofloxacin eye ointment was applied to the cornea.

### 4.8 Suprachoroidal Injection

Following ketamine/xylazine anesthesia, 2‐week‐old rats underwent a suprachoroidal injection procedure. Under stereoscopic guidance, a partial‐thickness scleral incision (80% depth) was made 1 mm posterior to the limbus using a 31‐gauge needle. A 33‐gauge beveled blunt needle (45° angle) attached to a 10 µL Hamilton syringe was then carefully advanced through the remaining scleral fibers into the suprachoroidal space. The cavity was expanded by gradual infusion of AAV8.hRPE65p.hTYRco vector (5 µL total volume containing 6 × 10^9^ GC, 2 × 10^10^ GC, or 6 × 10^10^ GC). The needle remained in place for 30 s postinjection before withdrawal. Subsequently, eye ointment was applied to the cornea. The rats were placed on a heating pad before awakening and returned to their cages.

### 4.9 Electroretinogram

Retinal function was evaluated by scotopic/photopic ERG (Phoenix Ganzfeld system) following 16 h dark adaptation. After ketamine/xylazine anesthesia with temperature maintenance, electrodes were placed (tail ground, forehead reference). Scotopic ERG of mice used six intensity levels (−1.1, 0.1, 0.7, 1.0, 1.3, and 2.2 log cd·s/m^2^), rats used five intensity levels (−1.1, 0.1, 0.7, 1.0 and 1.3 log cd·s/m^2^); photopic ERG employed green/UV stimuli (0.3, 0.9, 1.5, 2.1, 3, and 3.9 log cd·s/m^2^) after 5 min light adaptation (1.3 log cd/m^2^ background)

### 4.10 Optical Coherence Tomography

The OCT images of mice and rats were acquired using the HRA+OCT system (Heidelberg Engineering, Germany). The ONL thickness was assessed with a 30° lens. Measurements were taken at four inferior and four superior locations, positioned 0.5, 1.0, 2.0, and 3.0 mm from the optic disc, using the OCT system's built‐in software. The mean thickness at each location was derived from measurements in four orientations: clockwise 45°, counterclockwise 45°, vertical, and horizontal.

### 4.11 H&E and Masson–Fontana Staining

Enucleated eyes were fixed in 4% paraformaldehyde at room temperature for at least 6 h, dehydrated in ethanol series, cleared in xylene, and embedded in paraffin. The paraffin‐embedded retinas were cut into 5 µm‐thick sections for H&E or Masson–Fontana staining according to standard protocols. Images were collected with the pathological scanner (Pannoramic MIDI, 3DHISTECN, Hungary).

### 4.12 Visual Cliff Test

Visual function was evaluated using a modified visual cliff test, adapted from a previous study [50]. The apparatus comprised two plastic boxes, both 50 cm tall and 40 cm wide. The first box featured a covered upper section and an open lower half. A checkerboard pattern of black and white squares (2 cm × 2 cm each) adorned the covered portion, continuing downward onto the exposed half. The second box, positioned atop the first, had a transparent floor, an upward‐facing opening, and black surrounding walls (Figure 2D). In this setup, the second chamber functioned as an open arena divided into two equal sections: the first section, featuring a checkerboard‐patterned upper region, was designated as the “safe zone,” while the second section, containing a cliff between the transparent floor and the checkerboard base, was termed the “unsafe zone.” The apparatus maintained uniform illumination at 10 lux, measured at the eye level of the mice.

During step‐down trials, a raised platform was positioned between the checkerboard‐patterned and transparent sections to simulate a visual cliff. Each mouse underwent 10 trials, with their movement direction recorded as either a safe choice (toward the checkerboard‐patterned zone) or an unsafe choice (toward the transparent zone). All decisions were systematically documented and analyzed

### 4.13 Quantitative Analysis of Melatonin Production

The average optical density value was used to represent the amount of melanin produced. ImageJ was used to analyze the average optical density values of the cells or eyeballs, and all the images were inverted during the analysis. The lower the value is, the more melanin is produced.

### 4.14 Statistics

All statistical analyses were conducted using GraphPad Prism 9. Data are presented as mean ± SEM. Significance was determined by Dunnett's test (as specified in the figure legends), with *p* < 0.05 considered statistically significant.

## Author Contributions

Yang Yang and Fanfei Liu offered the main direction and significant guidance of this manuscript. Li Song and Qingnan Wang constructed the plasmid vectors. Fanfei Liu, Ming Hu, and Xiu Jin performed the cell culture and transfection. Li Song and Qingnan Wang produced the AAV8 vector and endotoxin assays. Li Song, Jing Su, Qin Ye, and Qiuxia Xu performed the mouse studies. Li Song and Manjun Li performed the rat studies. Yifang An, Qiqi Li, Manjun Li, and Xiaoyi Wu performed the western blot. Kaiqin She, Jiamei Fu, and Manjun Li performed the immunohistochemistry. Li Song and Chengda Ren drafted the manuscript. Yang Yang, Fang Lu, and Chengda Ren edited the manuscript. All listed authors have agreed to the final submitted version.

## Conflicts of Interest

Y. Y. and Q. W. are inventors on a patent application related to this work filed by Genevector Therapeutics, Inc. (PCT/CN2024/127561). Q. W. is a full‐time employee of Genevector Therapeutics, Inc., which provided assistance in data collection, including raising the animals involved in the study and provided the instrumentation required for ERG testing of the animals in this study. The remaining authors declare no conflicts of interest.

## Ethics Statement

The Institutional Animal Care and Concern Committee of Sichuan University provided approval for the animal experiments (20220221077), and the committee's rules were followed when caring for the animals.

## Supporting information




**Figure S1. Codon optimization enhances the expression of the *TYR* gene and melanin deposition. (A)** Representative cell pellet images of the HEK293 cells transiently transfected. **(B)** Analysis of the average optical density of the cell pellets (n = 3 in each group). The data are shown as the mean ± SEM. One‐way ANOVA and post hoc Dunnett's test were used for the comparison. ***p < 0.001. **(C)** Representative western blot analysis. Cell lysates were prepared from the cell pellet (a) for the detection of tyrosinase. GAPDH (36 kDa) was used as a loading control. **(D)** Grey value analysis of the western blot (*n* = 3 in each group). The data are shown as the mean ± SEM. One‐way ANOVA and post hoc Dunnett's test for the comparison. *p < 0.05.

## Data Availability

All relevant data supporting this study are contained within the manuscript and supplementary materials. Additional datasets may be obtained from the corresponding authors upon reasonable request.

## References

[1] R. A. King , D. Townsend , W. Oetting , et al., “Temperature‐sensitive Tyrosinase Associated With Peripheral Pigmentation in Oculocutaneous Albinism,” Journal of Clinical Investigation 87, no. 3 (1991): 1046–1053. 1900307 10.1172/JCI115064 PMC329899

[2] W. S. Oetting and R. A. King , “Molecular Basis of Oculocutaneous Albinism,” Journal of Investigative Dermatology 103, no. 5 (1994): 131S–136S. 7963676 10.1111/1523-1747.ep12399447

[3] R. A. King , J. Pietsch , J. P. Fryer , et al., “Tyrosinase Gene Mutations in Oculocutaneous Albinism 1 (OCA1): Definition of the Phenotype,” Human Genetics 113, no. 6 (2003): 502–513. 13680365 10.1007/s00439-003-0998-1

[4] V. S. Ramalingam , R. Sinnakirouchenan , and D. M. Thappa , “Malignant Transformation of Actinic Keratoses to Squamous Cell Carcinoma in an Albino,” Indian Journal of Dermatology 54, no. 1 (2009): 46–48. 20049269 10.4103/0019-5154.48986 PMC2800870

[5] M. Chaki , M. Sengupta , A. Mukhopadhyay , et al., “OCA1 in Different Ethnic Groups of India Is Primarily due to Founder Mutations in the Tyrosinase Gene,” Annals of Human Genetics 70, no. 5 (2006): 623–630. 16907708 10.1111/j.1469-1809.2006.00247.x

[6] P. S. Page‐McCaw , S. C. Chung , A. Muto , et al., “Retinal Network Adaptation to Bright Light Requires Tyrosinase,” Nature Neuroscience 7, no. 12 (2004): 1329–1336. 15516923 10.1038/nn1344

[7] M. F. Naso , B. Tomkowicz , W. L. Perry 3rd , et al., “Adeno‐Associated Virus (AAV) as a Vector for Gene Therapy,” Biodrugs 31, no. 7 (2017): 317–334. 28669112 10.1007/s40259-017-0234-5 PMC5548848

[8] D. Wang , P. W. L. Tai , and G. Gao , “Adeno‐associated Virus Vector as a Platform for Gene Therapy Delivery,” Nature Reviews Drug Discovery 18, no. 5 (2019): 358–378. 30710128 10.1038/s41573-019-0012-9 PMC6927556

[9] A. Pupo , A. Fernández , S. H. Low , et al., “AAV Vectors: The Rubik's cube of human Gene Therapy,” Molecular Therapy 30, no. 12 (2022): 3515–3541. 36203359 10.1016/j.ymthe.2022.09.015 PMC9734031

[10] S. S. Issa , A. A. Shaimardanova , V. V. Solovyeva , et al., “Various AAV Serotypes and Their Applications in Gene Therapy: An Overview,” Cells 12, no. 5 (2023): 785. 36899921 10.3390/cells12050785 PMC10000783

[11] H. C. J. Ertl , “Immunogenicity and Toxicity of AAV Gene Therapy,” Frontiers in Immunology 13 (2022): 975803. 36032092 10.3389/fimmu.2022.975803 PMC9411526

[12] J. H. Wang , D. J. Gessler , W. Zhan , et al., “Adeno‐associated Virus as a Delivery Vector for Gene Therapy of Human Diseases,” Signal Transduction and Targeted Therapy 9, no. 1 (2024): 78. 38565561 10.1038/s41392-024-01780-w PMC10987683

[13] A. Srivastava , “AAV Vectors: Are They Safe?,” Human Gene Therapy 31, no. 13–14 (2020): 697–699. 32611206 10.1089/hum.2020.187

[14] A. Gargiulo , C. Bonetti , S. Montefusco , et al., “AAV‐mediated Tyrosinase Gene Transfer Restores Melanogenesis and Retinal Function in a Model of Oculo‐cutaneous Albinism Type I (OCA1),” Molecular Therapy 17, no. 8 (2017): 1347–1354. 10.1038/mt.2009.112 PMC2835246 19436266

[15] T. D. Lamb and E. N. Pugh , “Dark Adaptation and the Retinoid Cycle of Vision,” Progress in Retinal and Eye Research 23, no. 3 (2004): 307–380. 15177205 10.1016/j.preteyeres.2004.03.001

[16] D. A. Thompson and A. Gal , “Vitamin A Metabolism in the Retinal Pigment Epithelium: Genes, Mutations, and Diseases,” Progress in Retinal and Eye Research 22, no. 5 (2003): 683–703. 12892646 10.1016/s1350-9462(03)00051-x

[17] J. R. Sparrrow , D. Hicks , and C. P. Hamel , “The Retinal Pigment Epithelium in Health and Disease,” Current Molecular Medicine 10, no. 9 (2010): 802–823. 21091424 10.2174/156652410793937813 PMC4120883

[18] M. Istrate , B. Vlaicu , M. Poenaru , et al., “Photoprotection Role of Melanin in the human Retinal Pigment Epithelium. Imaging Techniques for Retinal Melanin,” Romanian Journal of Ophthalmology 64, no. 2 (2020): 100–104. 32685774 PMC7339703

[19] A. Naylor , A. Hopkins , N. Hudson , et al., “Tight Junctions of the Outer Blood Retina Barrier,” International Journal of Molecular Sciences 21, no. 1 (2019): 211. 31892251 10.3390/ijms21010211 PMC6981689

[20] M. Kaufmann and Z. Han , “RPE Melanin and Its Influence on the Progression of AMD,” Ageing Research Reviews 99 (2024): 102358. 38830546 10.1016/j.arr.2024.102358 PMC11260545

[21] T. Taubitz , A. V. Tschulakow , M. Tikhonovich , et al., “Ultrastructural Alterations in the Retinal Pigment Epithelium and Photoreceptors of a Stargardt Patient and Three Stargardt Mouse Models: Indication for the central Role of RPE Melanin in Oxidative Stress,” PeerJ 6 (2028): e5215. 10.7717/peerj.5215 PMC6054867 30038866

[22] L. Hong and J. D. Simon , “Current Understanding of the Binding Sites, Capacity, Affinity, and Biological Significance of Metals in Melanin,” Journal of Physical Chemistry B 111, no. 28 (2007): 7938–7947. 17580858 10.1021/jp071439h PMC2533804

[23] S. R. Kim , Y. P. Jang , S. Jockusch , et al., “The *All‐trans* ‐retinal Dimer Series of Lipofuscin Pigments in Retinal Pigment Epithelial Cells in a Recessive Stargardt Disease Mode,” Proceedings National Academy of Science USA 104, no. 49 (2007): 19273–19278. 10.1073/pnas.0708714104 PMC2148280 18048333

[24] M. M. LaVail , R. L. Sidman , and C. O. Gerhardt , “Congenic Strains of RCS Rats With Inherited Retinal Dystrophy,” Journal of Heredity 66, no. 4 (1975): 242–244. 1172515 10.1093/oxfordjournals.jhered.a108621

[25] S. P. Sundelin , S. E. Nilsson , and U. T. Brunk , “Lipofuscin‐formation in Cultured Retinal Pigment Epithelial Cells Is Related to Their Melanin Content,” Free Radical Biology and Medicine 30, no. 1 (2001): 74–81. 11134897 10.1016/s0891-5849(00)00444-5

[26] K. Ding , J. Shen , Z. Hafiz , et al., “AAV8‐vectored Suprachoroidal Gene Transfer Produces Widespread Ocular Transgene Expression,” Journal of Clinical Investigation 129, no. 11 (2019): 4901–4911. 31408444 10.1172/JCI129085 PMC6819121

[27] I. C. Han , J. L. Cheng , E. R. Burnight , et al., “Retinal Tropism and Transduction of Adeno‐Associated Virus Varies by Serotype and Route of Delivery (Intravitreal, Subretinal, or Suprachoroidal) in Rats,” Human Gene Therapy 31, no. 23‐24 (2020): 1288–1299. 32948113 10.1089/hum.2020.043 PMC7757705

[28] W. M. Blaszczyk , L. Arning , K. P. Hoffmann , et al., “A Tyrosinase Missense Mutation Causes Albinism in the Wistar Rat,” Pigment Cell & Melanoma Research 18, no. 2 (2005): 144–145. 10.1111/j.1600-0749.2005.00227.x 15760344

[29] W. Xiong , D. M. Wu , Y. Xue , et al., “AAV *cis*‐Regulatory Sequences Are Correlated With Ocular Toxicity,” Proceedings National Academy of Science USA 116, no. 12 (2019): 5785–5794. 10.1073/pnas.1821000116 PMC6431174 30833387

[30] Z. Habot‐Wilner , G. Noronha , and C. C. Wykoff , “Suprachoroidally Injected Pharmacological Agents for the Treatment of Chorio‐retinal Diseases: A Targeted Approach,” Acta Ophthalmol 97, no. 5 (2019): 460–472. 30702218 10.1111/aos.14042

[31] L. Naftali Ben Haim and E. Moisseiev , “Drug Delivery via the Suprachoroidal Space for the Treatment of Retinal Diseases,” Pharmaceutics 13, no. 7 (2021): 967. 34206925 10.3390/pharmaceutics13070967 PMC8309112

[32] E. Touchard , M. Berdugo , P. Bigey , et al., “Suprachoroidal Electrotransfer: A Nonviral Gene Delivery Method to Transfect the Choroid and the Retina without Detaching the Retina,” Molecular Therapy 20, no. 8 (2012): 1559–1570. 22252448 10.1038/mt.2011.304 PMC3412485

[33] G. Yiu , S. H. Chung , I. N. Mollhoff , et al., “Suprachoroidal and Subretinal Injections of AAV Using Transscleral Microneedles for Retinal Gene Delivery in Nonhuman Primates,” Molecular Therapy—Methods & Clinical Development 16 (2020): 179–191. 32055646 10.1016/j.omtm.2020.01.002 PMC7005511

[34] E. Greggio , E. Bergantino , D. Carter , et al., “Tyrosinase Exacerbates Dopamine Toxicity but Is Not Genetically Associated With Parkinson's Disease,” Journal of Neurochemistry 93, no. 1 (2005): 246–256. 15773923 10.1111/j.1471-4159.2005.03019.x

[35] J. Huang , Y. X. Bai , S. W. Han , et al., “A Human TERT C‐Terminal Polypeptide Sensitizes HeLa Cells to H_2_O_2_‐Induced Senescence Without Affecting Telomerase Enzymatic Activity,” Biochemical and Biophysical Research Communications 301, no. 3 (2003): 627–632. 12565825 10.1016/s0006-291x(02)03049-8

[36] A. L. McCormack , M. Thiruchelvam , A. B. Manning‐Bog , et al., “Environmental Risk Factors and Parkinson's Disease: Selective Degeneration of Nigral Dopaminergic Neurons Caused by the Herbicide Paraquat,” Neurobiology of Disease 10, no. 2 (2002): 119–127. 12127150 10.1006/nbdi.2002.0507

[37] T. Hasegawa , M. Matsuzaki , A. Takeda , et al., “Increased Dopamine and Its Metabolites in SH‐SY5Y Neuroblastoma Cells That Express Tyrosinase,” Journal of Neurochemistry 87, no. 2 (2003): 470–475. 14511124 10.1046/j.1471-4159.2003.02008.x

[38] F. Giordano , C. Bonetti , E. M. Surace , et al., “The Ocular Albinism Type 1 (OA1) G‐Protein‐Coupled Receptor Functions With MART‐1 at Early Stages of Melanogenesis to Control Melanosome Identity and Composition,” Human Molecular Genetics 18, no. 23 (2009): 4530–4545. 19717472 10.1093/hmg/ddp415

[39] G. Raposo and M. S. Marks , “Melanosomes — Dark Organelles Enlighten Endosomal Membrane Transport,” Nature Reviews Molecular Cell Biology 8, no. 10 (2007): 786–797. 17878918 10.1038/nrm2258 PMC2786984

[40] V. M. Lopez , C. L. Decatur , W. D. Stamer , et al., “L‐DOPA Is an Endogenous Ligand for OA1,” PLoS Biology 6, no. 9 (2008): e236. 18828673 10.1371/journal.pbio.0060236 PMC2553842

[41] T. Burgoyne , R. Jolly , B. Martin‐Martin , et al., “Expression of OA1 Limits the Fusion of a Subset of MVBs With Lysosomes – A Mechanism Potentially Involved in the Initial Biogenesis of Melanosomes,” Journal of Cell Science 127, no. 3 (2014): 700. 10.1242/jcs.128561 PMC3828590 24006264

[42] V. Vachiramon and K. Thadanipon , “Postinflammatory Hypopigmentation,” Clinical and Experimental Dermatology 36, no. 7 (2011): 708–714. 21671990 10.1111/j.1365-2230.2011.04088.x

[43] X. Tian , Z. Cui , S. Liu , et al., “Melanosome Transport and Regulation in Development and Disease,” Pharmacology & Therapeutics 219 (2021): 107707. 33075361 10.1016/j.pharmthera.2020.107707

[44] O. Kholmanskikh , N. van Baren , F. Brasseur , et al., “Interleukins 1α and 1β Secreted by Some Melanoma Cell Lines Strongly Reduce Expression of MITF‐M and Melanocyte Differentiation Antigens,” International Journal of Cancer 127, no. 7 (2010): 1625–1636. 20099279 10.1002/ijc.25182

[45] C. Q. F. Wang , Y. T. Akalu , M. Suarez‐Farinas , et al., “IL‐17 and TNF Synergistically Modulate Cytokine Expression While Suppressing Melanogenesis: Potential Relevance to Psoriasis,” Journal of Investigative Dermatology 133, no. 12 (2013): 2741–2752. 23732752 10.1038/jid.2013.237 PMC3830693

[46] V. T. Natarajan , P. Ganju , A. Singh , et al., “IFN‐γ Signaling Maintains Skin Pigmentation Homeostasis Through Regulation of Melanosome Maturation,” Proceedings of National Academy of Sciences 111, no. 6 (2014): 2301–2306. 10.1073/pnas.1304988111 PMC3926048 24474804

[47] J. Zhou , J. Ling , Y. Wang , et al., “Cross‐Talk Between Interferon‐Gamma and Interleukin‐18 in Melanogenesis,” Journal of Photochemistry and Photobiology B: Biology 163 (2016): 133–143. 27567084 10.1016/j.jphotobiol.2016.08.024

[48] J. Zhou , J. Ling , and F. Ping , “Interferon‐γ Attenuates 5‐Hydroxytryptamine‐Induced Melanogenesis in Primary Melanocyte,” Biological & Pharmaceutical Bulletin 39, no. 7 (2016): 1091–1099. 27374284 10.1248/bpb.b15-00914

[49] M. Lock , M. Alvira , L. H. Vandenberghe , et al., “Rapid, Simple, and Versatile Manufacturing of Recombinant Adeno‐Associated Viral Vectors at Scale,” Human Gene Therapy 21, no. 10 (2010): 1259–1271. 20497038 10.1089/hum.2010.055 PMC2957274

[50] C. Felgerolle , B. Hébert , M. Ardourel , et al., “Visual Behavior Impairments as an Aberrant Sensory Processing in the Mouse Model of Fragile X Syndrome,” Frontiers in Behavioral Neuroscience 13 (2019): 228. 31680892 10.3389/fnbeh.2019.00228 PMC6797836

