# Supplementary material for: Retinal Pigment Epithelium‐Targeting Gene Therapy Corrects Ocular Symptoms in Mouse and Rat Models of Oculocutaneous Albinism Type I
Source: MedComm (2020). 2025 Oct 20;6(11):e70433. doi: 10.1002/mco2.70433 (PMC12536886; doi:10.1002/mco2.70433)
Supplement: Supplementary file 1 — Figure S1. Codon optimization enhances the expression of the TYR gene and melanin deposition. (A) Representative cell pellet images of the HEK293 cells transiently transfected. (B) Analysis of the average optical density of the cell pellets (n = 3 in each group). The data are shown as the mean ± SEM. One‐way ANOVA and post hoc Dunnett's test were used for the comparison. ***p < 0.001. (C) Representative western blot analysis. Cell lysates were prepared from the cell pellet (a) for the detection of tyrosinase. GAPDH (36 kDa) was used as a loading control. (D) Grey value analysis of the western blot (n = 3 in each group). The data are shown as the mean ± SEM. One‐way ANOVA and post hoc Dunnett's test for the comparison. *p < 0.05. [file MCO2-6-e70433-s001.docx]

Supplementary Materials for

Retinal pigment epithelium-targeting gene therapy corrects ocular symptoms in mouse and rat models of Oculocutaneous albinism Type Ⅰ

Li Song^2#^, Chengda Ren^1#^, Min Luo^2#^, Jing Su^2^, Xiu Jin^2^, Jiamei Fu^2^, Qiuxia Xu^2^, Xiaoyi Wu^2^, Fanfei Liu^1^, Qin Ye^2^, Man Liu^2^, Qiqi Li^2^, Yifang An^2^, Qingnan Wang^3^, Kaiqin She^1^, Fang Lu^1*^, Yang Yang^1,2^*

^1^ Department of Ophthalmology, West China Hospital, Sichuan University, Chengdu, Sichuan, China

^2^ State Key Laboratory of Biotherapy and Cancer Center, West China Hospital, Sichuan University and Collaborative Innovation Center, Chengdu, Sichuan, China

^3^ Chengdu Genevector Therapeutics, Inc., Chengdu, China

*Corresponding authors:

Fang Lu

Postal address: Guoxue xiang, No. 37, Chengdu, Sichuan, China 610041

Telephone number: +86 189 8060 2062

Email: lufang@wchscu.cn

Yang Yang

Postal address: Ke-yuan Road 4, No. 1, Gao-peng Street, Chengdu, Sichuan, 610041, China

Tel: + 86 028 85164063

Email: yang2012@scu.edu.cn


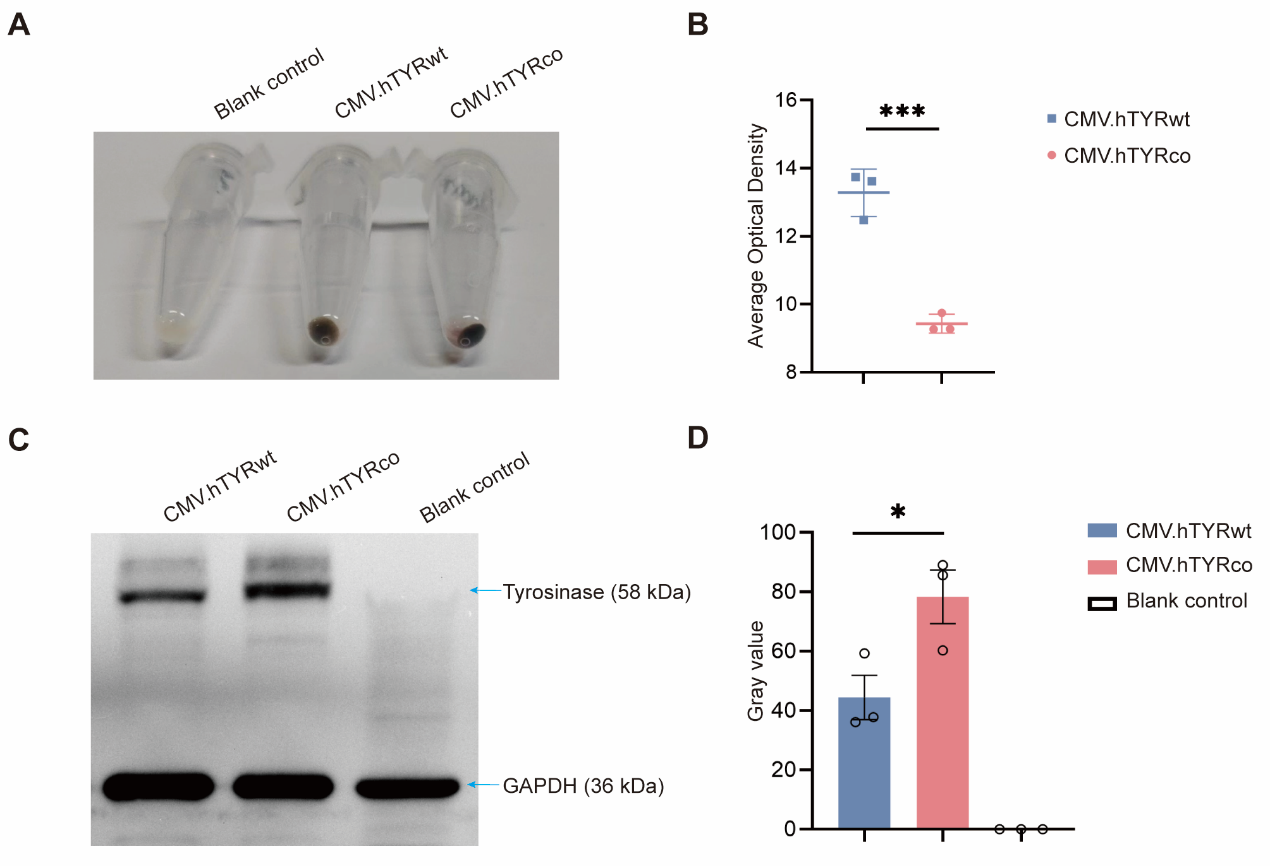
**Figure. S1. Codon optimization enhances the expression of *TYR* gene and melanin deposition. (A)** Representative cell pellets images of the HEK293 cells transiently transfected. **(B)** Analysis of the average optical density of the cell pellets (n=3 in each group). The data are shown as the mean ± SEM. One-way ANOVA and *post hoc* Dunnett’s test were used for the comparison. ***P<0.001. **(C)** Representative western blot analysis. Cell lysates were prepared from cell pellet (a) for detection of tyrosinase. GAPDH (36 kDa) was used as a loading control. **(D)** Grey value analysis of the western blot (n=3 in each group). The data are shown as the mean ± SEM. One-way ANOVA and *post hoc* Dunnett’s test for the comparison. *p<0.05.


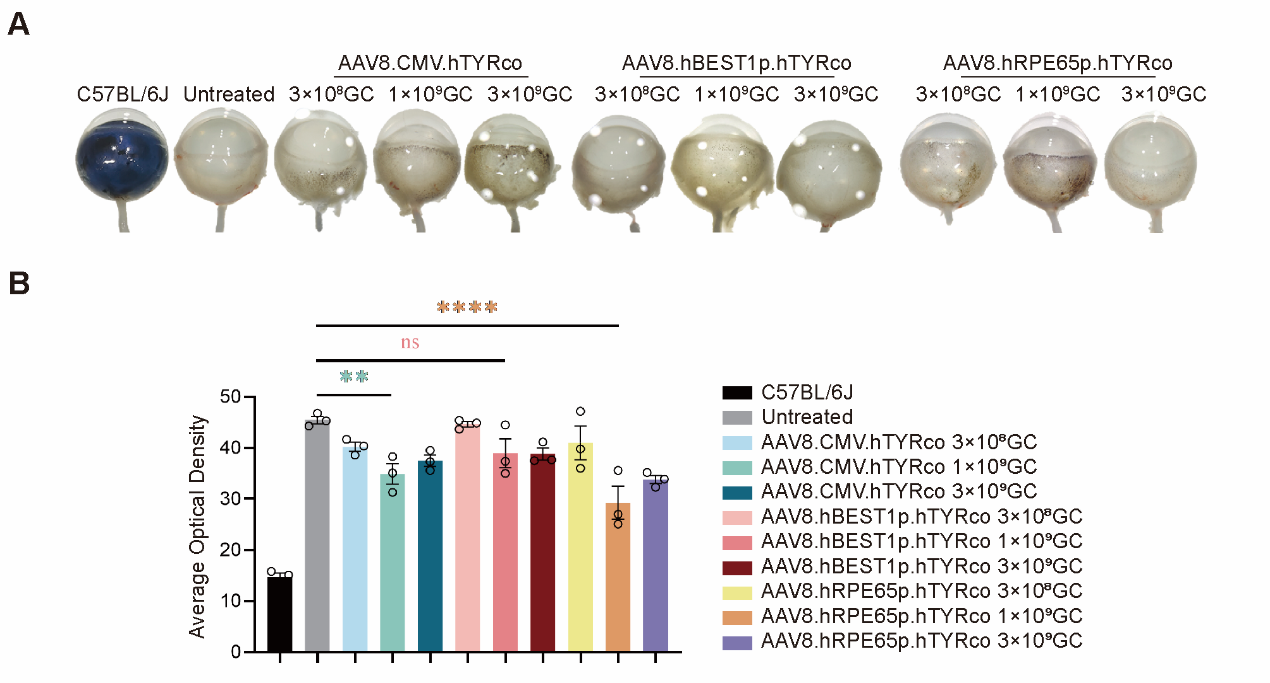
**Figure S2. Gene therapy promoted the biosynthesis of melanin.** **(A)** Representative eye images of the mice at 3 months post-injection. **(B)** Analysis of the average optical density of eye images of the mice at 3 months post-injection (n=3 in each group). The data are shown as the mean ± SEM. One-way ANOVA and *post hoc* Dunnett’s test was used for comparisons with the untreated group. *p<0.05, **p<0.01, ***p<0.001, ****p<0.0001, ns, nonsignificant difference.

**
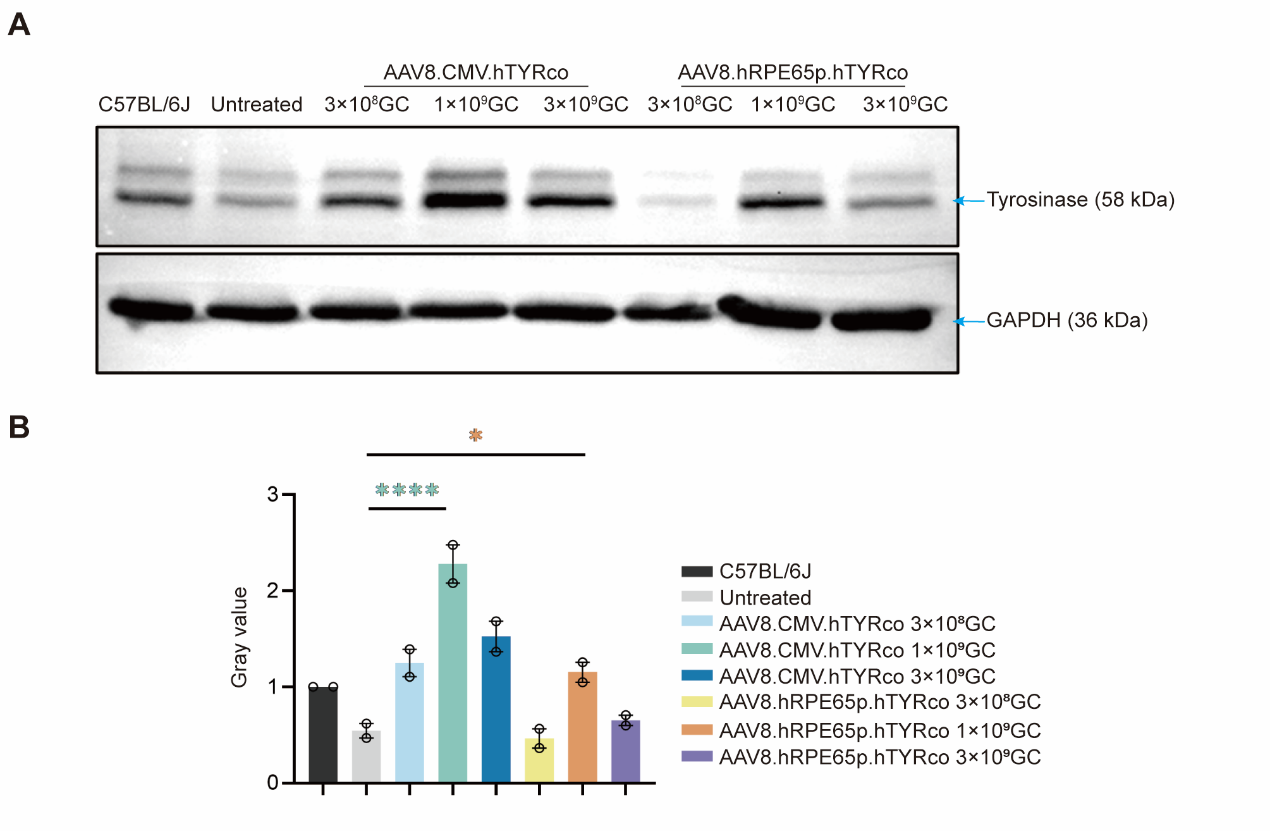
Figure S3. Gene therapy restored the expression of functional tyrosinase protein in mice. (A)** Representative western blot analysis of WT and untreated and treated B6 albino mice eyes at 12 months post-injection. GAPDH (36 kDa) was used as a loading control. **(B)** Gray value analysis of the western blot in **(A)**. The data are shown as the mean ± SEM. One-way ANOVA with *post hoc* Dunnett’s test was used for comparison with the untreated group (n=2 in each group). *P<0.05, **P<0.01, ***P<0.001, ****P<0.0001.


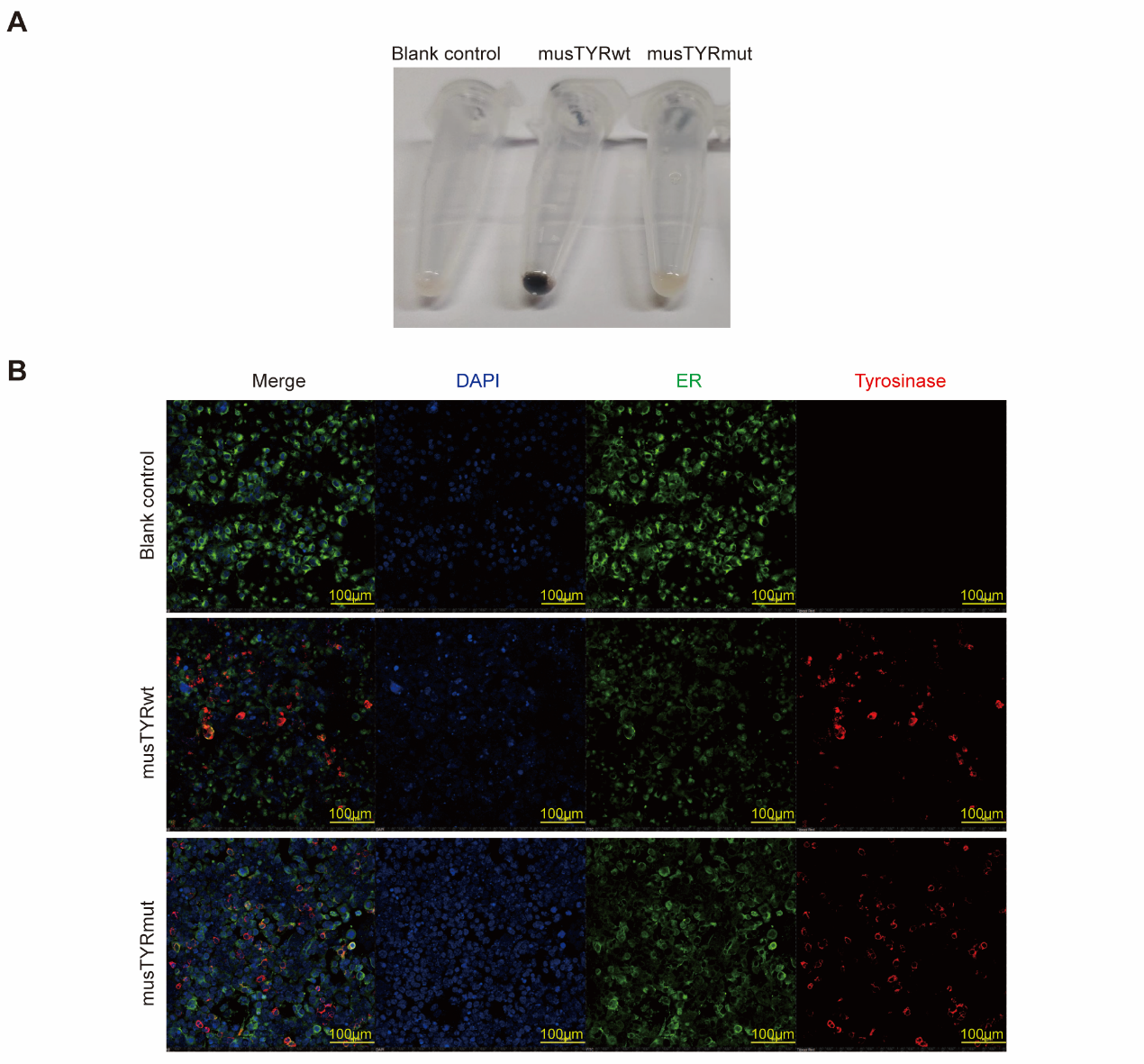
**Figure S4.** **Vectors constructed in vitro containing *TYR* gene of wild-type mice or B6 albino mice, and verifying whether their expression of tyrosinase is functional. (A)** Cell pellets of the HEK293 cells transfected transiently. Blank control: HEK293 cells without transfection; musTYRwt: wild-type mice; musTYRmut: B6 albino mice. **(B)** Immunofluorescence staining was performed on the cells collected in **(A)**. Nuclei were labeled with DAPI (blue); Endoplasmic reticulum are labeled with calnexin (green); Red represents tyrosinase.


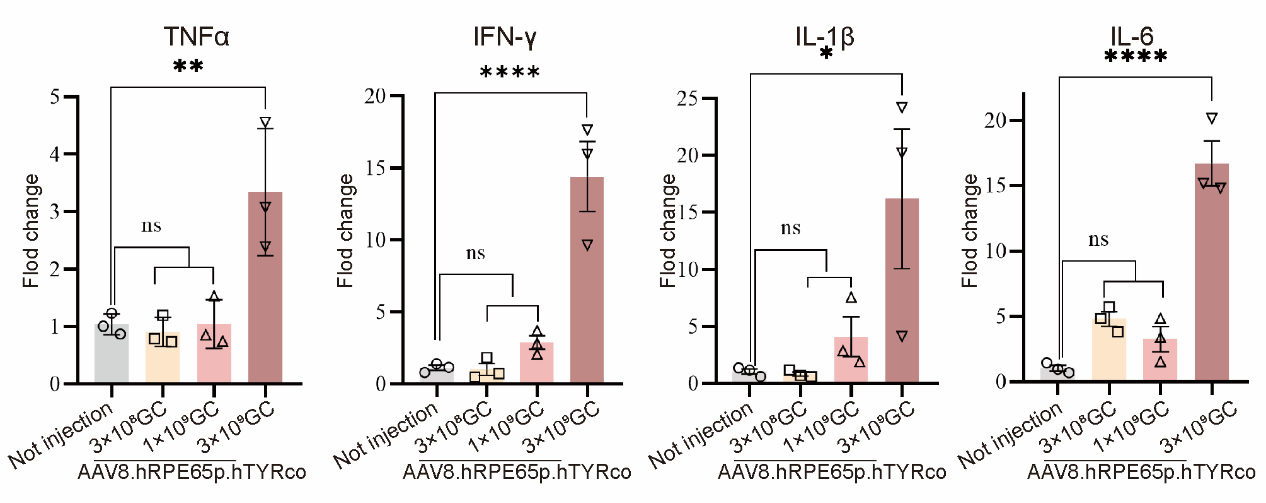
**Figure S5.** **Relative mRNA levels of TNF-α, IL-1β, IL-6, and IFN-γ by qPCR in the retinas of treated mice at 12 months post-injection with AAV8.hRPE65p.hTYRco.** Expression level was normalized to *GAPDH*. The data are shown as the mean ± SEM (n=3 of each group). One-way ANOVA with *post hoc* Dunnett’s test was used for comparison with the uninjection group. *P<0.05, **P<0.01, ***P<0.001, ****P<0.0001, ns, nonsignificant difference.


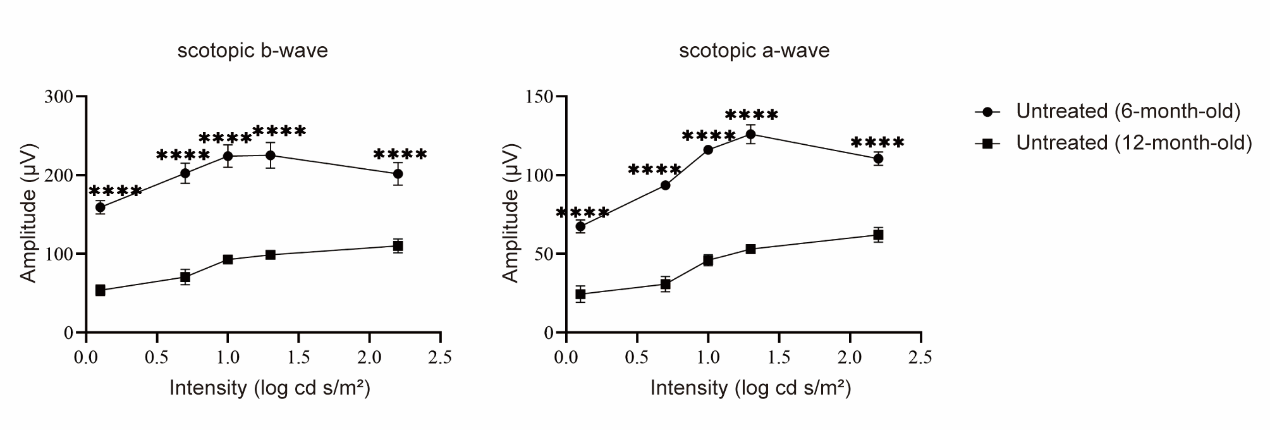
**Figure S6. ERG** **b- and a-wave amplitudes of B6 albino mice at different ages under scotopic.** The data are shown as the mean ± SEM (n=6, each group). Two-way ANOVA and *post hoc* Dunnett’s test was used for the comparison. ****P<0.0001.


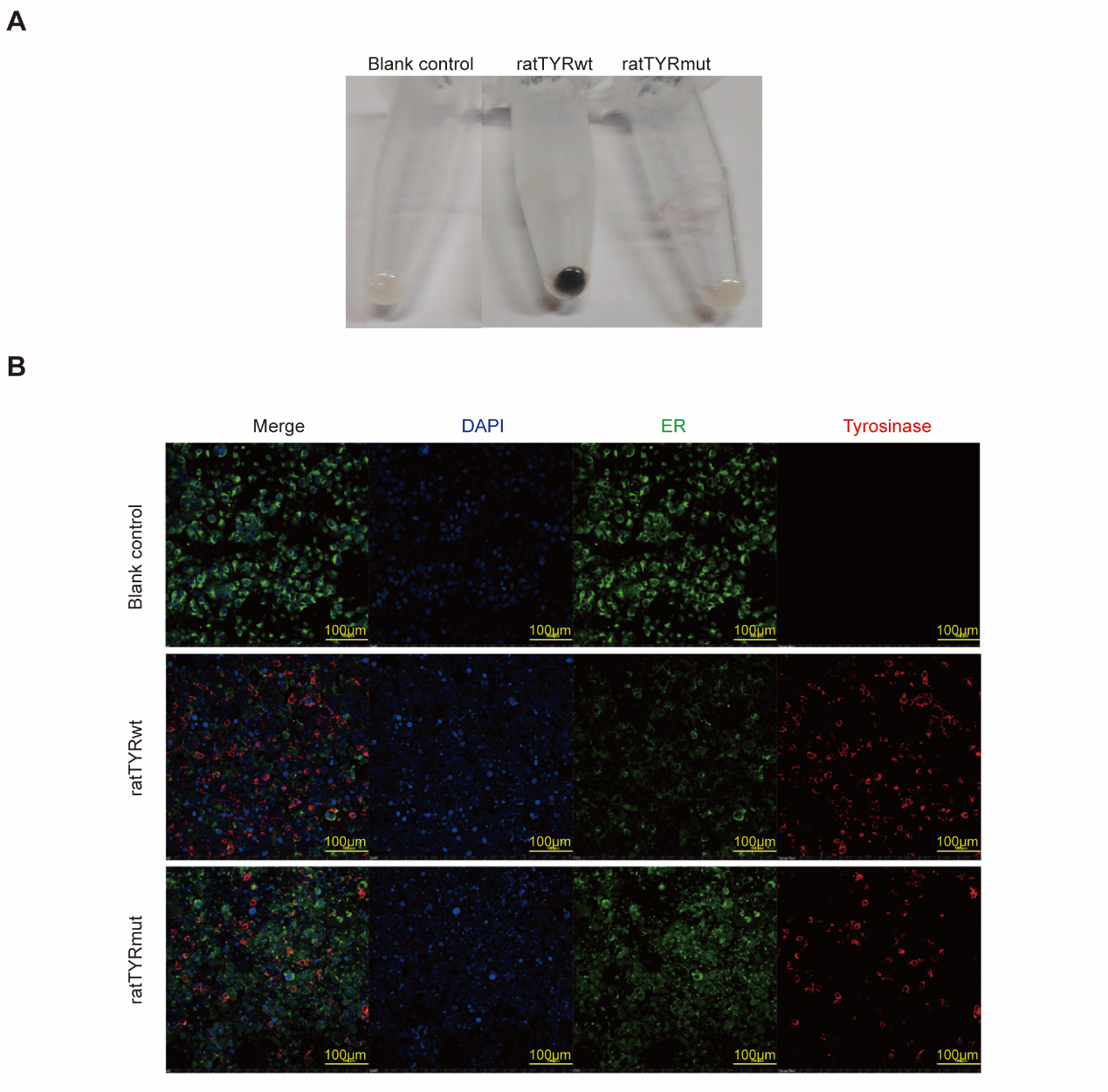
**Figure S7. Vectors constructed in vitro containing *TYR* gene of wild-type rat or WISTAR rat, and verifying whether their expression of tyrosinase is functional. (A)** The cell pellets of the HEK293 cells transfected transiently. Blank control: HEK293 cells without transfection; ratTYRwt: wild-type rat; ratTYRmut: WISTAR rat. **(B)** Immunofluorescence staining was performed on the cells collected in **(A)**. Nuclei was labeled with DAPI (blue); Endoplasmic reticulum was labeled with calnexin (green); Red represents tyrosinase.


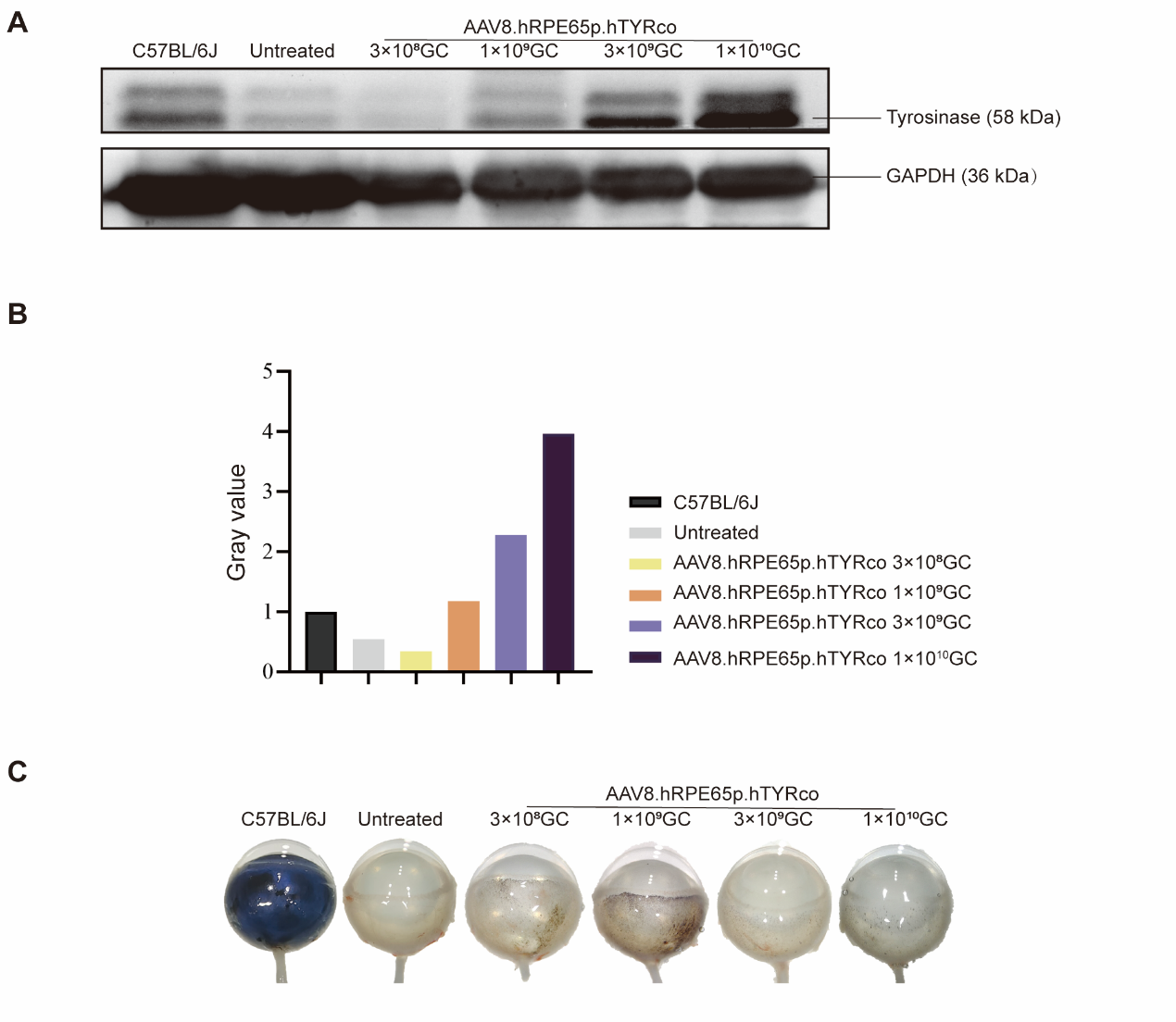
**Figure S8. Detection the expression of tyrosinase in mice treated with AAV8.hRPE65p.hTYRco at different doses. (A)** Western blot analysis of WT mice and untreated and treated mice with AAV8.hRPE65p.hTYRco at 3 months post-injection. GAPDH (36 kDa) was used as a loading control. **(B)** Gray value analysis of the western blot in (**A**). **(C)** Representative eye images of mice in **(A)**.
